# A Pro‐Metastatic Derivatives Eliminator for In Vivo Dual‐Removal of Circulating Tumor Cells and Tumor‐Derived Exosomes Impedes their Biodistribution into Distant Organs

**DOI:** 10.1002/advs.202304287

**Published:** 2023-10-22

**Authors:** Ying Sun, Lei Xing, Jun Luo, Ming‐Tao Yu, Xiao‐Jie Wang, Yi Wang, Tian‐Jiao Zhou, Hu‐Lin Jiang

**Affiliations:** ^1^ State Key Laboratory of Natural Medicines China Pharmaceutical University Nanjing 210009 China; ^2^ Jiangsu Key Laboratory of Druggability of Biopharmaceuticals China Pharmaceutical University Nanjing 210009 China; ^3^ Jiangsu Key Laboratory of Drug Discovery for Metabolic Diseases China Pharmaceutical University Nanjing 210009 China

**Keywords:** biodistribution, circulating tumor cells, dual removal, hemoperfusion, metastatic cascade, tumor‐derived exosomes

## Abstract

Circulating tumor cells (CTCs) and tumor‐derived exosomes (TDEs) play an irreplaceable role in the metastatic cascade and preventing them from reaching distant organs via blood circulation helps to reduce the probability of cancer recurrence and metastasis. However, technologies that can simultaneously prevent CTCs and TDEs from reaching distant organs have not been thoroughly developed until now. Here, inspired by hemoperfusion, a pro‐metastatic derivative eliminator (PMDE) is developed for the removal of both CTCs and TDEs from the peripheral blood, which also inhibits their biodistribution in distant organs. This device is designed with a dual antibody‐modified immunosorbent filled into a capture column that draws peripheral blood out of the body to flow through the column to specifically capture CTCs and TDEs, followed by retransfusing the purified blood into the body. The PMDE can efficiently remove CTCs and TDEs from the peripheral blood and has excellent biocompatibility. Interestingly, the PMDE device can significantly inhibit the biodistribution of CTCs and TDEs in the lung and liver by scavenging them. This work provides a new perspective on anti‐metastatic therapy and has broad prospects in clinical applications to prevent metastasis and recurrence.

## Graphical abstract

CTCs and TDEs are organotropic to reach specific distant organs through blood circulation. TDEs are like fertilizers to make the soil (pre‐metastatic niche) fertile for seed (CTCs) growth. The proposed pro‐metastatic derivatives eliminator (PMDE) can inhibits the biodistribution of CTCs and TDEs to distant organs by efficiently capturing and removing them from peripheral blood.

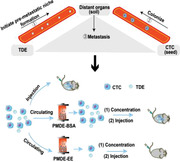

## 1 Introduction

**Figure 1:**
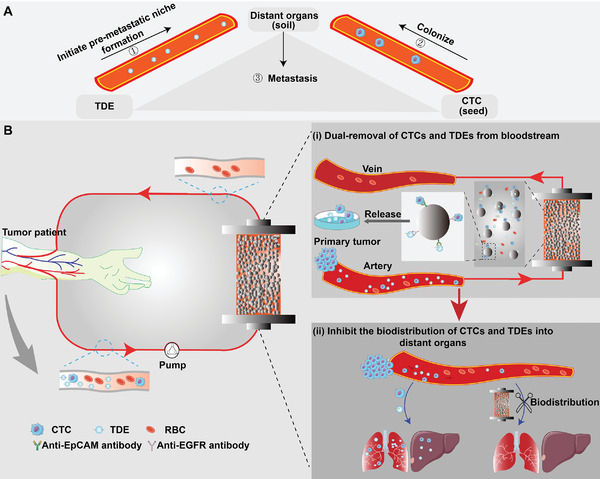
A) A schematic diagram of the triangular relationship among TDEs, CTCs, and metastasis. B) Diagram illustrating the mechanism of the pro‐metastatic derivatives eliminator.

Tumor metastasis is the cause of ≈90% of cancer‐related deaths, with poor therapeutic options.^[^ ^1^ ^]^ Hematogenous metastasis is the predominant method of metastasis.^[^ ^2^ ^]^ Circulating tumor cells (CTCs) shed from the primary tumor into the peripheral blood circulation, evade recognition by the immune system, and infiltrate into distant organs to form metastases.^[^ ^3^ ^]^ The number of CTCs in the peripheral blood of tumor patients is closely related to cancer metastasis and recurrence.^[^ ^4^ ^]^ Notably, increasing evidence shows that tumor‐derived exosomes (TDEs, small extracellular vesicles secreted by tumor cells that are 30–150 nm in size) are also responsible for initiating tumor metastasis.^[^ ^5^ ^]^ TDEs act as messengers sent into blood circulation by the primary tumor and reach specific distant organs to prepare a pre‐metastatic niche for CTC colonization even before their arrival.^[^ ^6^ ^]^ The “seed and soil” hypothesis suggests that CTCs (seeds) colonize in the pre‐metastatic niche (soil) to facilitate tumor metastasis.^[^ ^2^, ^6^ ^]^ TDEs act as fertilizers to make the soil (pre‐metastatic niche) fertile for seeds (CTCs) growth. Earlier studies have shown that CTCs and TDEs are organotropic to reach specific distant organs through the blood circulation to form metastases (**Figure** **1**A).^[^ ^5^, ^7^ ^]^ In short, in the metastatic cascade, both CTCs and TDEs are indispensable to jointly initiate metastasis formation. Unfortunately, after tumor patients receive traditional anti‐tumor therapy such as surgical resection, radiotherapy, or chemotherapy, the counts of CTCs and TDEs in the bloodstream tend to increase, allowing them to reach distant organs via the blood circulation and increase the risk of tumor metastasis and recurrence.^[^ ^8^ ^]^ Hence, the key to solving this problem is to prevent CTCs and TDEs from being distributed to distant organs. Until now, there has been no well‐established method to simultaneously prevent the biodistribution of CTCs and TDEs via blood circulation to distant organs. Therefore, we wondered whether we could simultaneously eliminate CTCs and TDEs in the bloodstream to prevent their biodistribution in distant organs.

Recently, technologies for in vivo CTC removal have been developed. For example, injectable tumor‐targeting magnetic nanomaterials that sufficiently interact with CTCs in vivo are commonly used for CTC removal.^[^ ^9^ ^]^ Although this strategy opens a way for CTCs to be removed by nanomaterials, there are still issues to be addressed, including the low stability in blood, uncontrollable aggregation, and non‐specific cellular internalization of the nanomaterials. To overcome these problems, great scientific efforts have been committed to the development of an integrated trapped device (ITD) for local therapy to avoid systemic toxicity. Such an ITD can selectively eliminate CTCs from the bloodstream or specifically enrich and completely damage CTCs in blood vessels by antibody‐modified ITD, such as indwelling needles, 3D scaffold structures, and intravenous catheters.^[^ ^4^, ^10^ ^]^ Nevertheless, the efficacy of inhibiting the biodistribution of CTCs and TDEs in distant organs with these technologies has not been proven. Moreover, these studies have focused only on the removal of CTCs and have neglected the irreplaceable role of TDEs in tumor metastasis. Therefore, strategies for dual‐removal of CTCs and TDEs from the peripheral blood and inhibiting their biodistribution to distant organs with high biosafety are promising for mitigating or even preventing metastasis.

Among these technologies for enriching CTCs or TDEs, the general problem that remains is that the capture efficiency for CTCs and TDEs is low when only the anti‐EpCAM antibody is used as the capture antibody.^[^ ^11^ ^]^ The generally accepted reasons are that CTCs often lose EpCAM expression by undergoing phenotypic changes during epithelial‐mesenchymal transition (EMT).^[^ ^12^ ^]^ Therefore, to improve the capture efficiency of CTCs and TDEs, a mixture of cancer cell membrane antibodies, such as anti‐EpCAM and anti‐EGFR antibodies, was conjugated onto the surfaces of substrates.^[^ ^11a,b^ ^]^ Although CTCs and TDEs are pro‐metastatic derivatives, they can be utilized as tumor samples for noninvasive diagnosis.^[^ ^13^ ^]^ In recent years, studies have been conducted to isolate and detect CTCs and TDEs with methods such as those utilizing immunomagnetic beads,^[^ ^14^ ^]^ micro/nano substrates,^[^ ^11^, ^15^ ^]^ and microfluidic chips.^[^ ^16^ ^]^ However, typical blood volumes (5–10 mL) for in vitro analysis account for only approximately one‐thousandth of the total amount of blood in the body, which may show sampling bias, leading to false negative results.^[^ ^10b^ ^]^ Ideally, a strategy for dual‐enrichment of CTCs and TDEs with improved capture efficiency from all of the blood in the body can overcome this challenge.

Hemoperfusion is a widely used medical treatment in blood purification technology. The principle of hemoperfusion is to draw human blood out of the body by establishing an extracorporeal circulation branch to flow through a hemoperfusion device containing adsorbents to remove toxins by utilizing the binding of toxins and the adsorbent.^[^ ^17^ ^]^ Herein, inspired by hemoperfusion, we developed a pro‐metastatic derivative eliminator (PMDE) for dual‐removal of CTCs and TDEs from the peripheral blood circulation to impede their biodistribution in distant organs. The PMDE device consisted of immunosorbents functionalized with two tumor‐specific antibodies, a capture column, intravenous catheters, indwelling needles, and a peristaltic pump. The immunosorbent was prepared by coupling anti‐EpCAM and anti‐EGFR antibodies with Sepharose microspheres, which have excellent hemocompatibility and are commonly used hemoperfusion materials, and that can be easily modified with crosslinkers duo to the large number of hydroxyl groups.^[^ ^18^ ^]^ The principle of this device is to draw the peripheral blood out of the body into an established an extracorporeal circulation branch so that the blood flows through the capture column containing immunosorbents to simultaneously remove CTCs and TDEs by exploiting the binding of CTCs and TDEs to the immunosorbents surface. Moreover, the captured CTCs and TDEs, as tumor samples, are released to provide materials for downstream analysis (“turning waste into treasure”) (Figure 1B). Additionally, we performed in vitro and in vivo safety assessments of the PMDE and confirmed its outstanding biocompatibility and hemocompatibility. Overall, the PMDE device presented here has potential clinical applications for anti‐metastatic therapy, as it can lower the burden of CTCs and TDEs in tumor patients and prevent their biodistribution in distant organs.

## 2 Results and Discussion

### 2.1 Tumor‐Specific Antibody Functionalization of Sepharose Microspheres

**Figure 2:**
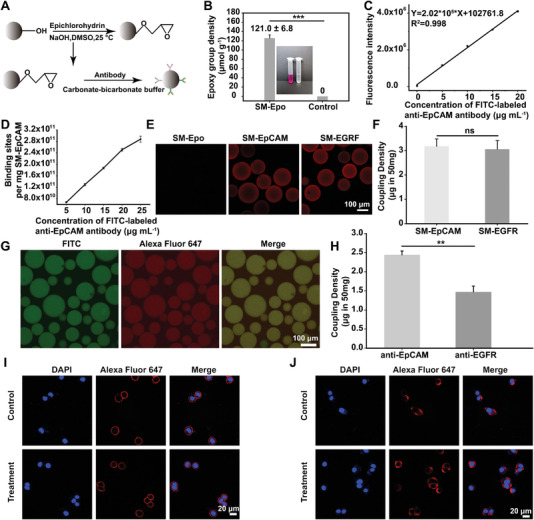
The characterization and verification of sepharose microspheres functionalized with tumor‐specific antibody. A) Schematic illustration of tumor‐specific antibody coupled with SMs. B) Optimal epoxy group density of SMs‐Epo after reacting with Epichlorohydrin (*n* = 3, the control is zero). C) The linear relationship between the concentrations of FITC‐labeled anti‐EpCAM antibody and fluorescence intensity. D) Estimated binding site density of CTCs and TDEs on sepharose microsphere surfaces coated with FITC‐labeled anti‐EpCAM antibody (*n* = 3). E) SMs‐Epo was incubated with anti‐EpCAM antibody and anti‐EGFR antibody, respectively, then with Alexa Fluor 647‐labeled goat anti‐mouse IgG. Scale bar: 100 µm. F) There is no difference in coupling density between SMs‐EpCAM and SMs‐EGFR. G) Fluorescence image of SMs‐EE (Alexa Fluor 647‐labeled anti‐EpCAM antibody and FITC‐labeled anti‐EGFR antibody were used). Scale bar: 100 µm. H) The coupling density of anti‐EpCAM and anti‐EGFR antibody on SMs‐EE. I,J) The biorecognition activity of I) anti‐EpCAM antibody and J) anti‐EGFR antibody after overnight coupling reaction in the carbonate‐bicarbonate buffer. Scale bar: 20 µm. Data were shown as mean ± SD. *p <0.05, **p <0.01, ***p <0.001 and ns: not significant.

According to the antibody coupling route (**Figure** **2**A), Sepharose microspheres (SMs) with an average particle size of 124.0 ± 2.7 µm (Figure S1, Supporting Information) were first epoxidized, and the greatest epoxidation efficiency was 121.0 ± 6.8 µmol g^−1^ after optimization of the reaction conditions (Figure 2B; Figure S2, Supporting Information). Within a certain range, the density of epoxy groups increased as more epichlorohydrin (ECH) was added, and the maximum epoxy group density was reached when the concentration of epichlorohydrin was 10%. The optimal concentration of NaOH was 0.4 m, whereas at higher concentrations, hydrolysis of the epoxy group caused a decrease in epoxy group density. The color difference between SMs grafted with epoxy groups (SMs‐Epo) and SMs without epoxy groups was evident after reaction with 3 mL of Na_2_S_2_O_3_ (1.3 m in ultrapure water) and 100 µL of phenolphthalein (inset of Figure 2B). These results demonstrated that the SMs were successfully epoxidized. The principle of the color development reaction is shown in Figure S3 (Supporting Information).

Antibody coupling reaction was then carried out via a ring‐opening reaction between the epoxy groups of SMs‐Epo and the amino group of antibody in carbonate buffer. To examine the antibody coupling density and efficiency of SMs‐Epo, FITC‐labeled anti‐EpCAM antibody was first used to establish a standard fluorescence curve and fluorescence spectrograms (Figure 2C; Figure S4, Supporting Information). The antibody coupling density (C_D_) and efficiency (C_E_) were calculated by Equation (1) and Equation (2), respectively.

(1) $$\begin{array}{ccc} C_{D} & = & \frac{(C_{1}-C_{2})\cdot V}{M_{g}}\;\left(\mu g/\mathrm{mg}\right)\;\;\mathrm{or}\;\;\\ C_{D} & = & \frac{n\cdot NA}{M_{g}}\;\;\left(\mathrm{binding}\;\mathrm{sites}/\mathrm{mg}\right) \end{array}$$

(2) $$\;C_{E}=\frac{C_{1}-C_{2}}{C_{1}}\;\;$$

Here, C_1_ (µg mL^−1^) is the initial concentration of the fluorescent antibody; C_2_ is the residual concentration of the fluorescent antibody, which was calculated according to the standard curve; V (mL) is the volume of fluorescent antibody added; Mg (mg) is the weight of the SMs; *n* (mol) is the molar amount of the coupled antibody; and NA is Avogadro's constant. The results indicated that the higher the concentration of anti‐EpCAM antibody was, the higher the antibody density was (Figure 2D; Figures S5 and S6, Supporting Information). However, the coupling efficiency slightly decreased from 33.20 ± 1.12% to 28.30 ± 1.04% (Figure S7, Supporting Information), indicating that more of the antibody was dissociated and had not coupled with SMs‐Epo. This may be related to the fewer epoxy groups compared to the number of antibodies. Thus, accounting for the cost and coupling density, an antibody concentration of 20 µg mL^−1^ was chosen for subsequent experiments. Finally, a coupling density of 3.18 ± 0.30 µg per 50 mg of SMs‐Epo (Figure S6, Supporting Information) was achieved at an antibody concentration of 20 µg mL^−1^, which demonstrated that there was an equivalent to 2.54 × 10^11^ binding sites per milligram of SMs‐EpCAM that can bind CTCs and TDEs (Figure 2D) according to Equation (1). Theoretically, compared with previous studies, this immobilized antibody density is sufficiently high to mediate the immunorecognition among the CTCs, TDEs, and tumor‐specific antibodies grafted on the SMs‐Epo.^[^ ^11b^ ^]^ Similarly, a standard curve of FITC‐labeled anti‐EGFR antibody was established (Figure S8, Supporting Information) to investigate the coupling density and efficiency of the anti‐EGFR antibody. The SMs‐Epo were coupled with an anti‐EpCAM or anti‐EGFR antibody before being recognized and imaged using Alexa Fluor 647‐labeled goat anti‐mouse IgG. The results showed that the fluorescence intensities of both samples were essentially the same (Figure 2E; Figure S9, Supporting Information). Quantitative analysis further indicated that when the same concentration of antibody (20 µg mL^−1^) was added, the coupling density was 3.06 ± 0.36 µg per 50 mg of SMs‐Epo (2.45 × 10^11^ binding sites per milligram), which was similar to that of the anti‐EpCAM antibody (Figure 2F). Next, SMs‐Epo coupled with both anti‐EpCAM and anti‐EGFR antibodies (SMs‐EE) was investigated. The results indicated that anti‐EpCAM and anti‐EGFR antibodies could be successfully coupled with SMs‐Epo (Figure 2G). Further quantitative analysis showed that the coupling density of the anti‐EpCAM antibody was slightly higher than that of the anti‐EGFR antibody (Figure 2H). The antibody coupling densities of the anti‐EpCAM and anti‐EGFR antibodies were 2.44 ± 0.10 µg and 1.47 ± 0.15 µg per 50 mg of SMs‐EE, respectively, which was equivalent to 1.95 × 10^11^ anti‐EpCAM antibody binding sites and 1.18 × 10^11^ anti‐EGFR antibody binding sites per milligram of SMs‐EE that can bind CTCs and TDEs according to Equation (1). To investigate the capture efficiency of SMs coupled with different antibodies, four different SMs were prepared (Figure 2G; Figure S10, Supporting Information), including SMs‐BSA (SMs coupled with BSA only as a negative control), SMs‐EpCAM (SMs coupled with anti‐EpCAM antibody only), SMs‐EGFR (SMs coupled with anti‐EGFR antibody only), and SMs‐EE (SMs coupled with both anti‐EpCAM antibody and anti‐EGFR antibodies). Since the coupling reaction was carried out under alkaline conditions, we specifically examined whether the antibody still maintained antigen‐binding activity after the overnight coupling reaction in carbonate buffer, thus we examined whether the anti‐EpCAM and anti‐EGFR antibodies could bind effectively to the corresponding antigens. The immunofluorescence assay revealed that the antibodies were able to bind well to the corresponding antigen on the cell membrane of MDA‐MB‐468 cells, maintaining their original antigen binding activity (Figure 2I,J). In brief, the anti‐EpCAM and anti‐EGFR antibodies can be coupled with SMs‐Epo simultaneously with high antibody density, and the antibodies maintain their original antigen binding activity. This facilitate biorecognition and binding between CTCs, TDEs, and immunosorbent.

### 2.2 In Vitro Static Capture of CTCs and TDEs

**Figure 3:**
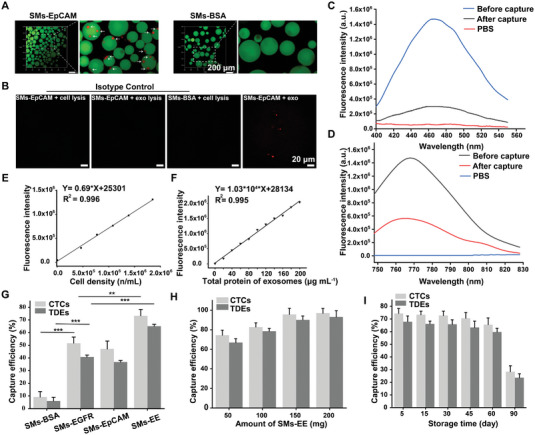
The capture of cancer cells and TDEs under static conditions. A) The 3D fluorescence images of MDA‐MB‐468 cells prestained with DiD captured on SMs‐EpCAM (FITC labeled anti‐EpCAM antibody was used). Scale bar: 200 µm. B) Fluorescence images showing red spots on SMs‐EpCAM‐exosome samples, reflecting the binding of Alexa Fluor® 647‐labeled goat anti‐rabbit IgG to CD81, thus the presence of exosomes only in this sample compared to the three negative controls. Scale bar: 20 µm. C) Fluorescence spectra of DAPI‐stained MDA‐MB‐468 cell suspensions before and after capture by SMs‐EE. D) Fluorescence spectra of DiR‐stained MDA‐MB‐468 cells‐derived exosome suspensions before and after capture. E) Standard curves between cell density and fluorescence intensity. F) Standard curves between exosome concentrations and fluorescence intensity. G) The capture efficiency of CTCs and TDEs captured by the four different functionalized SMs (*n* = 3). H) The capture efficiency as a function of the amount of SMs‐EE added (*n* = 3). I) The storage stability of SMs‐EE at 4 °C (*n* = 3). Data were shown as mean ± SD. *p <0.05, **p <0.01, ***p <0.001.

EpCAM‐positive MDA‐MB‐468 cells and EpCAM‐negative HeLa cells were selected as model CTCs and control cells, respectively. First, EpCAM and EGFR expression in both cell lines were examined by immunofluorescence assays (Figure S11, Supporting Information). The results indicated that MDA‐MB‐468 cells indeed highly expressed EpCAM and EGFR, while HeLa cells hardly expressed EpCAM or EGFR, which is consistent with previous studies.^[^ ^19^ ^]^ Notably, cell passage did not affect the expression of EpCAM or EGFR in MDA‐MB‐468 cells, indicating that capture efficiency was unaffected by cell passaging (Figure S12, Supporting Information). Moreover, the expression of EpCAM and EGFR in exosomes derived from these two cell lines was also investigated by Western blot assay. The results in Figure S13 (Supporting Information) show that the exosomal EpCAM and EGFR expression is consistent with that of their parent cells. Therefore, we chose MDA‐MB‐468 cell‐derived exosomes as model TDEs and those derived from HeLa cells as a negative control. To visualize the cancer cells captured on the surface of SMs‐EpCAM surface, 2 × 10^5^ MDA‐MB‐468 cells stained with DiD were added into a 2 mL EP tube with 50 mg of SMs‐EpCAM and then incubated at 37 °C for 1 h. As displayed in **Figure** **3**A, the confocal images (with z‐stock superimposed) showed significant capture of MDA‐MB‐468 cells by SMs‐EpCAM compared to SMs‐BSA. Subsequently, to examine the performance of SMs‐EpCAM in capturing TDEs, we performed an immunofluorescence assay using an antibody that specifically recognizes a commonly used exosome marker protein, transmembrane CD81. As shown in Figure 3B, a strong fluorescence signal was observed on the surface of SMs‐EpCAM when using a fluorescent antibody against CD81, while almost no fluorescence signal was observed in the other three isotype controls. Altogether, these results revealed that exosomes with intact vesicle structures can be specifically captured by an anti‐EpCAM antibody on the surface of SMs‐EpCAM. These fluorescent spots may be generated due to aggregation of fluorescently labeled exosomes, which is consistent with previous studies.^[^ ^20^ ^]^ Notably, we found that the fluorescence intensities of the DAPI‐stained cell suspensions and DiR‐stained exosome suspensions changed significantly before and after capture (Figure 3C,D), which indicated that the counts of CTCs and TDEs in the suspensions decreased significantly and further demonstrating that CTCs and TDEs were captured by the immunosorbents successfully.

Interestingly, there was an excellent linear relationship between the fluorescence intensity and concentrations of stained cancer cells or TDEs (Figure 3E,F; Figure S14, Supporting Information). Thus, this relationship was used as a quantitative tool for capture efficiency. The capture efficiency of CTCs and TDEs was calculated according to Equation (3) and Equation (4), respectively.

(3) $$\;C_{E}=\frac{N_{1}-N_{2}}{N_{1}}\;\;$$

(4) $$\;C_{E}=\frac{M_{1}-M_{2}}{M_{1}}\;\;$$

where C_E_ refers to the capture efficiency of CTCs or TDEs, N_1_ refers to the initial number of cancer cells added, and N_2_ refers to the count of dissociated cancer cells in the supernatant after capture, which was calculated based on the fluorescence intensity, volume, and the standard curve. M_1_ refers to the initial amount (µg) of TDEs added, and M_2_ refers to the amount of dissociated TDEs in the supernatant after capture, which was calculated based on the fluorescence intensity, the volume, and the standard curve. To verify the capture efficiency when adding CTCs and TDEs simultaneously, as shown in Figure S15 (Supporting Information), we added 2 × 10^5^ MDA‐MB‐468 cells stained with DAPI and 50 µg of MDA‐MB‐468 cell‐derived exosomes labeled with DiR simultaneously and incubated them with 50 mg of immunosorbents at 37 °C. The capture efficiency of cancer cells and TDEs by SMs‐EE showed a time‐dependent increase, which was optimal at 60 min (Figure S16, Supporting Information). We can see that the capture efficiency of exosomes is lower than that of cancer cells; therefore, perhaps more binding sites are required to capture exosomes compared to cancer cells. Next, the capture abilities of four different SMs (SMs‐BSA, SMs‐EpCAM, SMs‐EGFR, and SMs‐EE) were investigated. Compared to the SMs‐EpCAM and SMs‐EGFR, the SMs‐EE exhibited the highest capture efficiency, reaching 73.3 ± 4.9% and 65.1 ± 1.6% for cancer cells and TDEs, respectively (Figure 3G). Hence, it is necessary to enhance the capture efficiency by exploiting the bivalent interactions between antibodies and antigens on the surface of CTCs and TDEs with heterogeneous antigen expression, which is consistent with previous studies.^[^ ^11a,b^ ^]^ We then chose SMs‐EE for subsequent experiments. Notably, as the amount of SMs‐EE invested increased to 200 mg, the capture efficiency increased to 96.9 ± 4.8% and 93.1 ± 6.4% for cancer cells and TDEs, respectively (Figure 3H). This is related to the larger surface area when the amount of SMs‐EE increased. Therefore, to a certain extent, the capture efficiency can be adjusted and improved by increasing the amount of SMs‐EE. Then, the storage stability of the SMs‐EE was examined after storage in the dark at 4 °C for 90 days. We found that the fluorescence intensity of SMs‐EpCAM and SMs‐EGFR decreased slightly in the first 60 days (Figure S17, Supporting Information). Similarly, the capture efficiency decreased only slightly in the first 60 days (Figure 3I). The results indicated that the antibody was not shed from the SMs‐EE and maintained good biorecognition, proving that SMs‐EE had excellent storage stability for up to 60 days. This antibody coupling strategy may be productized for the immunosorbents. We can thus conclude that: i SMs coupled with tumor‐specific antibodies can successfully capture MDA‐MB‐468 cells and their exosomes, and SMs functionalized with a bivalent antibody had the highest capture ability; and ii) the capture efficiency was higher as more SMs‐EE were added. These works laid the foundation for the in vivo removal of both CTCs and TDEs.

### 2.3 Dual‐Removal of CTCs and TDEs Using the PMDE in a Closed‐Loop Circulation System

**Figure 4:**
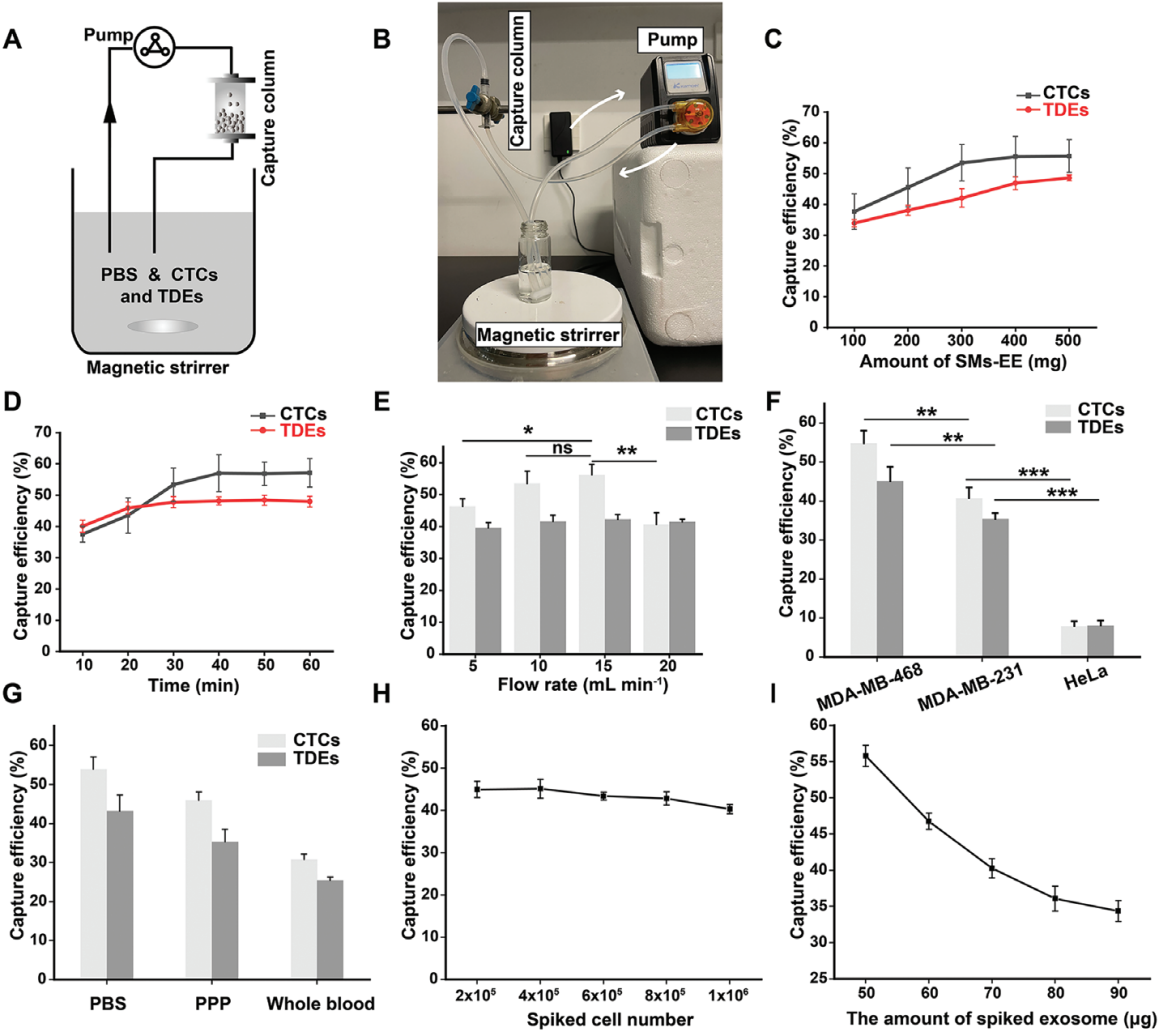
The removal of CTCs and TDEs by using PMDE in a closed‐loop circulation system. A) Schematic illustration of PMDE for dual‐removal CTCs and TDEs. B) Photograph of the simulated circulatory system. C‐E) The capture efficiency of cancer cells and TDEs as a function of C) the amount of SMs‐EE invested, D) circulating time, and E) flow rate (*n* = 3). F) The effect of the antigen expression level on capture efficiency (*n* = 3). G) The effect of different circulation media on capture efficiency (*n* = 3). H,I) The effect of interaction between cancer cells and TDEs on capture efficiency (*n* = 3). Data were shown as mean ± SD. *p <0.05, **p <0.01, ***p <0.001 and ns: not significant.

To simulate the practical application conditions of PMDE, we performed in vitro dynamic circulating experiments and further investigated the binding kinetics in a closed‐loop circulation system. (**Figure** **4**A,B). As we can see in the video (Movie S1, Supporting Information), the SMs‐EE were evenly dispersed in the capture column and moved rapidly. This ensured a high collision frequency between the SMs‐EE and CTCs and TDEs in circulation. Similar to the static capture experiments, the capture efficiencies of cancer cells and their exosomes increased as the amount of SMs‐EE put into the capture column increased, with the capture efficiency nearly peaking at 300 mg, reaching 53.5 ± 5.9% and 42.02 ± 3.0% for CTCs and TDEs, respectively (Figure 4C). The capture efficiency decreased significantly, compared to the static condition. This may be related to the fact that the CTCs and TDEs are subjected to large shear stress in the circulation system. To some extent, we can improve the capture efficiency of CTCs and TDEs by altering the amount of SMs‐EE invested in the capture column. Accounting for the capture efficiency and the cost, we chose 300 mg of SMs‐EE for subsequent experiments. We further examined the effect of circulation time on capture efficiency and found that the capture efficiency increased with circulation time (Figure 4D). The capture efficiency of cancer cells peaked at 30 min, while the capture efficiency of TDEs peaked sooner, which may be related to the higher collision frequency between exosomes and SMs‐EE. In addition, compared to static condition, the capture efficiency in the closed‐loop circulation system peaks in a shorter time. We can hypothesize that CTCs and TDEs have a higher probability of collision with SMs‐EEs in the closed‐loop circulation system with a flow rate of 10 mL min^−1^, which increases the likelihood that CTCs and TDEs would come into contact with SMs‐EE for specific biorecognition. We also examined the effect of flow rate on the capture efficiency and found that it had almost no effect on the capture efficiency of exosomes, while a large flow rate that was too high decreased the capture efficiency of tumor cells (Figure 4E). The reason for this may be that compared to TDEs, CTCs are subject to larger shear forces and more susceptible to changes in flow rate. Thus, both flow rates of 10 and 15 mL min^−1^ can be chosen. A rat model was chosen for the subsequent in vivo capture experiment, and the blood flow rate in rats is ≈10 mL min^−1^. Therefore, a flow rate of 10 mL min^−1^ was chosen for the subsequent study. To verify the objectivity of using SMs‐BSA as a negative control, since CD45 was negatively expressed during CTCs identification,^[^ ^21^ ^]^ we selected anti‐CD45 antibody to couple with the SMs to prepare SMs‐CD45 and found that both SMs‐BSA and SMs‐CD45 had no significant capture effect on tumor cells or TDEs, which proved that it was reasonable to use SMs‐BSA as a negative control (Figure S18, Supporting Information). To investigate the biospecificity of SMs‐EE to capture cancer cells and TDEs, three different cell lines and their exosomes, MDA‐MB‐468 cells (high EpCAM expression), MDA‐MB‐231 cells (low EpCAM expression), and HeLa cells (EpCAM‐negative cells), were spiked into the closed‐loop circulation system, respectively. The capture efficiencies of MDA‐MB‐468 cells and their exosomes (54.9 ± 3.2% and 45.1 ± 3.6%, respectively) were higher than those of MDA‐MB‐231 cells and their exosomes (40.7 ± 2.8% and 35.4 ± 1.4%, respectively) (Figure 4F). However, almost no HeLa cells nor their exosomes were captured by SMs‐EE. These results showed that SMs‐EE exhibited good biospecificity. Antigen expression in cancer cells and their exosomes significantly affects the binding affinity between antigens and antibodies on SMs‐EE.^[^ ^22^ ^]^ Next, we investigated the effect of the circulating medium and the interaction between MDA‐MB‐468 cells and their exosomes and found that the capture efficiency was the lowest when whole blood was used as the circulating medium, and the platelet‐poor plasma (PPP) was in the middle (Figure 4G). We inferred that the viscosity of the medium and the presence of many blood cells may impede the binding of CTCs and TDEs to the tumor‐specific antibody on the surface of the immunosorbent. Moreover, increasing the count of MDA‐MB‐468 cells slightly decreased the capture efficiency of tumor exosomes (Figure 4H), while increasing the initial amount of MDA‐MB‐468 cell‐derived exosomes significantly decreased the capture efficiency of cancer cells decreased significantly (Figure 4I). We can therefore infer that in the circulating capture system, CTCs and TDEs might compete for the binding sites. Overall, we further demonstrated the feasibility of using the PMDE to simultaneously remove CTCs and TDEs from the peripheral blood circulation.

### 2.4 Releasing Cancer Cells and TDEs Captured by the PMDE

**Figure 5:**
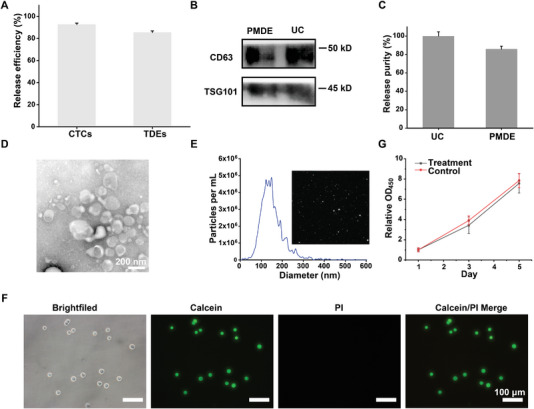
Release of captured CTCs and TDEs. A) The release efficiency of CTCs and TDEs (*n* = 3). B) Western blot analysis for exosomes released from PMDE or collected by UC (Ultracentrifugation). C) The relative purity of exosomes released from PMDE compared with exosomes collected by UC (*n* = 3). D) TEM images of exosomes released from SMs‐EE. E) The size and concentration of the released exosomes by NTA. The inset is an image showing the snapshot of video tracking (X_10_, 91.2 nm; X_50_, 139.5 nm; and, X_90_, 217.0 nm). F) Calcein and PI (Propidium Iodide) co‐staining of the released cancer cells. Scale bar: 100 µm. G) Captured cells remain viable for cell reculture and proliferate over a period of 5 days, as determined by a CCK‐8 assay normalized to the initial cell number (*n* = 3). Data were shown as mean ± SD.

Current technologies for cancer diagnosis targeting CTCs or TDEs suffer from sampling bias and possibly produce false negatives since these technologies process only approximately one‐thousandth of the total amount of blood in the body.^[^ ^16^ ^]^ In contrast, this PMDE has access to the entire peripheral blood supply, and the throughput of PMDE is much larger than that of the current technologies, reaching 600 mL h^−1^ (Table S1, Supporting Information), thus allowing the acquisition of representative tumor samples for diagnosis and detection. After circulating, we retrieved the SMs‐EE from the capture column and released the captured CTCs and TDEs using trypsin EDTA and pH 2.2 glycine‐HCl buffer, respectively. The release efficiencies reached 92.7 ± 1.2% and 85.6 ± 1.3% for cancer cells and TDEs, respectively (**Figure** **5**A). The purity of the released exosomes reached 85.9 ± 3.2% that of the gold standard method (ultracentrifugation) (Figure 5B,C). The details of the evaluation methodology are described in the experimental section of the Supporting Information. The morphology and size of the released exosomes were characterized by transmission electron microscope (TEM) (Figure 5D) and nanoparticle tracking analysis (NTA) (Figure 5E), and the video tracking of the released exosomes can be seen in Movie S2 (Supporting Information). These results showed that the exosomes maintained their intact structure and morphology and were not destroyed by the capture and release process. Moreover, the released MDA‐MB‐468 cells maintained good cell viability, as determined by calcein (green, live cells) and propidium iodide (PI) (red, dead cells) costaining (Figure 5F). Due to the rarity of CTCs, cell reculture could be performed to meet the needs of tumor diagnosis and detection, and the released cells were found to possess high proliferative ability compared with tumor cells without capture/release treatment by CCK‐8 assay (Figure 5G). Overall, it is feasible to use this PMDE to provide tumor samples for tumor diagnosis and detection by releasing the captured CTCs and TDEs with high efficiency. This PMDE is expected to compensate for the false negatives caused by other small sample detection technologies such as microfluidics.

### 2.5 Hemocompatibility and Biocompatibility Evaluations of the SMs‐EE

**Figure 6:**
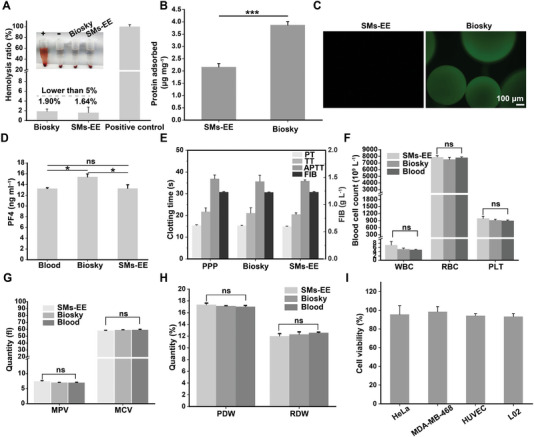
Blood compatibility and biocompatibility evaluation of SE‐MM. A) Haemolysis ratio and digital photos of SMs‐EE and Biosky (*n*  =  3). (+): positive control, deionized water; (−): negative control, normal saline. B) The protein adsorption amounts of SMs‐EE and Biosky (*n*  =  3). C) The fluorescence images show SMs‐EE and Biosky after incubation in FITC‐labeled HSA solution. Scale bar: 100 µm. D) Generated concentrations of PF4 after incubation of SMs‐EE and Biosky in whole blood (*n*  =  3). E) TT, APTT, PT, and FIB in PPP after incubation with SMs‐EE and Biosky, respectively. (*n*  =  3). F) Comparison of the blood cell count for normal whole blood and the blood after incubating with SMs‐EE and Biosky, respectively. (*n*  =  3, WBC: white blood cell count, RBC: red blood cell, PLT: platelet count). G,H) Platelet and red blood cell volume and volume distribution of whole blood and the blood after incubation with SMs‐EE and Biosky, respectively (*n* = 3, MPV: mean platelet volume, MCV: mean corpuscular volume, PDW: platelet distribution width, RDW: red cell distribution width). I) The cell viability of HeLa, MDA‐MB‐468, HUVEC, and L02 cells after incubation with SMs‐EE and Biosky by CCK‐8 assay (n = 6). Data were shown as mean ± SD. *p <0.05, **p <0.01, ***p <0.001 and ns: not significant.

To examine the clinical application potential of the PMDE, we investigated the hemocompatibility and biocompatibility of the SMs‐EE in vitro. First, the erythrocyte compatibility of SMs‐EE was investigated using hemolysis assays (**Figure** **6**A; Figure S19, Supporting Information). SMs‐EE showed a lower hemolysis rate (1.64 ± 1.1%) than Biosky (1.90 ± 0.47%) (a clinically applied hemoperfusion adsorbent from Bosin Biotechnology Co., Ltd.). This not only met the requirements of the hemolysis assay for an applied material (ASTM, F756‐2008) but also indicated better erythrocyte compatibility.^[^ ^23^ ^]^ After incubation and centrifugation, the supernatants of all groups containing different concentrations of SMs‐EE were colorless, and the erythrocytes had intact cell structures (Figure S20, Supporting Information). These results indicated that no hemoglobin was released and that the red blood cells did not rupture. Next, human serum albumin (HSA) was used to evaluate the plasma protein adsorption of the SMs‐EE. The results showed that compared to Biosky (3.87 ± 0.14 µg mg^−1^), the plasma protein adsorption of the SMs‐EE was lower, at only 2.16 ± 0.13 µg mg^−1^ (Figure 6B). The reason for this is that Biosky is synthesized from polystyrene divinylbenzene (PS‐DVB), which has strong hydrophobic adsorption, but Sepharose microspheres contain many hydroxyl groups and are extremely hydrophilic. The lower HSA adsorption capacity of SMs‐EE was further demonstrated by fluorescence imaging (Figure 6C), and these data indicated good hemocompatibility and that some undesirable effects, such as albumin loss and thrombus formation, could be reduced. Then, the effect of the SMs‐EE on platelet activation was investigated by measuring the platelet factor 4 (PF_4_) concentration (Figure 6D). After incubation with SMs‐EE, the PF4 concentrations were lower than those after incubation with Biosky and not significantly different from those after incubation in whole blood. This suggested that the SMs‐EE did not induce platelet activation because platelet activation releases PF_4_ when the biomaterial is exposed to blood. In addition, the coagulation properties of the SMs‐EE were investigated, including analyses of activated partial thromboplastin time (APTT), thrombin time (TT), prothrombin time (PT), and fibrinogen (FIB) (Figure 6E). APTT and TT can evaluate the in vitro antithrombogenicity of the samples; PT can confirm the exogenous coagulation ability, and FIB can evaluate the procoagulant activity.^[^ ^24^ ^]^ The APTT, TT, PT, and FIB values of the SMs‐EE did not change significantly compared with the PPP and Biosky groups, suggesting that the SMs‐EE almost did not cause whole blood coagulation. Subsequently, the effect of SMs‐EE on blood cells was investigated with an in vitro blood test. The results showed that SMs‐EE did not cause significant changes in the count of blood cells (Figure 6F) or their size (Figure 6G,H). Finally, the cytocompatibility of the SMs‐EE was further investigated using HeLa, MDA‐MB‐468, HUVEC, and L02 cells as models. It was found that the SMs‐EE did not affect the proliferative activity of these cells, as they maintained more than 90% of the proliferative activity (Figure 6I). Taken together, these results suggest that SMs‐EE are safe when in direct contact with blood, thus it is feasible to use SMs‐EE as a bivalent tumor‐specific antibody functionalized immunosorbent for dual‐removal of CTCs and TDEs from peripheral blood circulation.

### 2.6 Dual‐Removal of CTCs and TDEs in Living Animals and In Vivo Biosafety Assessment of the PMDE

**Figure 7:**
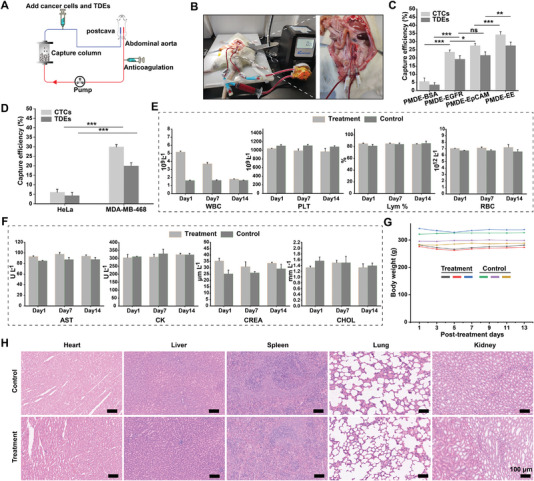
Dual‐removal of CTCs and TDEs in a rat model and in vivo biosafety evaluation of PMDE. A) schematic of in vivo circulating. B) Photograph of in vivo circulating in a rat model. C) The capture efficiency of CTCs and TDEs by the four different functionalized SMs in a rat model (*n* = 3). D) The specificity of PMDE to capture CTCs and TDEs in vivo (*n* = 3). E) Hematological analysis of the rats at days 1, 7, and 14 post‐treatment (*n* = 3, WBC: white blood cell count, PLT: platelet count, Lym: lymphocyte, RBC: red blood cell). F) Blood biochemical analysis of the rats at days 1, 7, and 14 post‐treatment (*n* = 3, AST: aspartate aminotransferase, CK: creatine kinase, CREA: creatinine, CHOL: cholesterol). G) Body weight of rats after surgery. H) H&E staining of heart, liver, spleen, lung, and kidney after surgery on day 14 (magnification: 20 ×). Scale bar: 100 µm. Data were shown as mean ± SD. *p <0.05, **p <0.01, ***p <0.001 and ns: not significant.

The in vitro study results demonstrated that the PMDE was efficient and safe for the simultaneous elimination of CTCs and TDEs. To further investigate the clinical application prospects of the PMDE, we performed in vivo circulation experiments and toxicity assessments in a rat model. Indwelling needles were transplanted into the abdominal aorta and postcava of rats to establish an in vivo circulation model (**Figure** **7**A,B). Then, 2 × 10^5^ MDA‐MB‐468 cells stained with DAPI and 50 µg of MDA‐MB‐468 cell‐derived exosomes stained with DiR were injected through the tee port of the catheter. PMDE‐BSA, in which the capture column was filled with SMs‐BSA, was used as a negative control group, and the efficiency of PMDE‐EpCAM (the capture column was filled with SMs‐EpCAM), PMDE‐EGFR (the capture column was filled with SMs‐EGFR), and PMDE‐EE (the capture column was filled with SMs‐EE) capture of CTCs and TDEs was investigated (Figure 7C). PMDE‐EE had a higher capture efficiency for MDA‐MB‐468 cells and their exosomes than that of PMDE‐EpCAM and PMDE‐EGFR (Figure 4F). However, the capture efficiency of PMDE‐EE did not increase exponentially. The fundamental factors affecting the capture efficiency mainly include the antibody coupling density on the surface of the substrate and the antigens expressed on the cell surface.^[^ ^11^, ^22^ ^]^ Since MDA‐MB‐468 cells highly express EpCAM and EGFR, the main factor affecting the capture efficiency is the antibody coupling density on the SMs‐EE. According to the results of the previous antibody coupling experiments (Figure 2F,H), although the total antibody density on the surface of SMs‐EE (3.13 × 10^11^ binding sites per milligram) is slightly higher than those of SMs‐EpCAM (2.54 × 10^11^ binding sites per milligram) and SMs‐EGFR (2.45 × 10^11^ binding sites per milligram), the antibody coupling densities of anti‐EpCAM (1.95 × 10^11^ binding sites per milligram) and anti‐EGFR antibody (1.18 × 10^11^ binding sites per milligram) on the surface of SMs‐EE was not exponentially higher than that of SMs‐EpCAM and SMs‐EGFR. In addition, the spatial distribution of the two antibodies on the SMs‐EE surface may result in less than optimal antibody utilization efficiency to capture CTCs and TDEs. Notably, the capture efficiency in vivo was lower than that in vitro dynamic circulation. It can be inferred from the above binding kinetic study that this result was related to medium viscosity and presence of many blood cells, which may impede the binding of CTCs and TDEs to the tumor‐specific antibody on the surface of the immunosorbent. In addition, normal exosomes in the bloodstream may block binding sites. Next, we used HeLa cells as a negative control and found that PMDE‐EE specifically removed CTCs and TDEs in vivo (Figure 7D), which is consistent with the above results in Figure 3F. Compared with all of the currently available methods for the in vivo removal of CTCs from peripheral blood circulation, the progressiveness of this PMDE is the significantly improved in vivo capture efficiency and efficient dual‐removal of CTCs and TDEs from the whole‐body blood (Table S2, Supporting Information). To further investigate the in vivo toxicity of the PMDE, a blood routine examination was first performed on days 1, 7, and 14 after circulating surgery (Figure 7E; Figure S21, Supporting Information). The results revealed an elevated white blood cell (WBC) count and a normal lymphocyte count in the early postoperative period, which suggested an inflammatory response that was mostly caused by surgery itself. After surgery, the WBC count recovered to normal, showing that the PMDE did not elicit a significant immunological response. In addition, there were no obvious changes in the other hematologic indexes. Then, the blood biochemical parameters, including liver function, total proteins, blood lipids, blood glucose, and renal function were analyzed on days 1, 7, and 14 after circulating surgery (Figure 7F; Figure S22, Supporting Information). No noticeable changes in the blood biochemical index were observed. During 14 days after surgery, we also monitored the body weights of the rats (Figure 7G). The results showed that the rats showed slight weight loss after surgery, which might be related to the the small amount of blood that was lost during surgery. However, the body weights recovered in a short time. On day 14, the heart, liver, spleen, lung, and kidney were sliced and stained with hematoxylin and eosin (H&E) (Figure 7H). These results showed no obvious harmful effects on the five major organs. Thus, these results demonstrate that the PMDE had no observable toxicity or side effects. Overall, this PMDE effectively eliminated CTCs and TDEs in the bloodstream and did not cause significant toxicity in vivo. Future studies can focus on improving the in vivo capture efficiency, such as optimizing the shape of the capture column to increase the collision frequency and increasing the amount of immunosorbent invested in the capture column.

### 2.7 PMDE Inhibited the Biodistribution of CTCs and TDEs in Distant Organs

**Figure 8:**
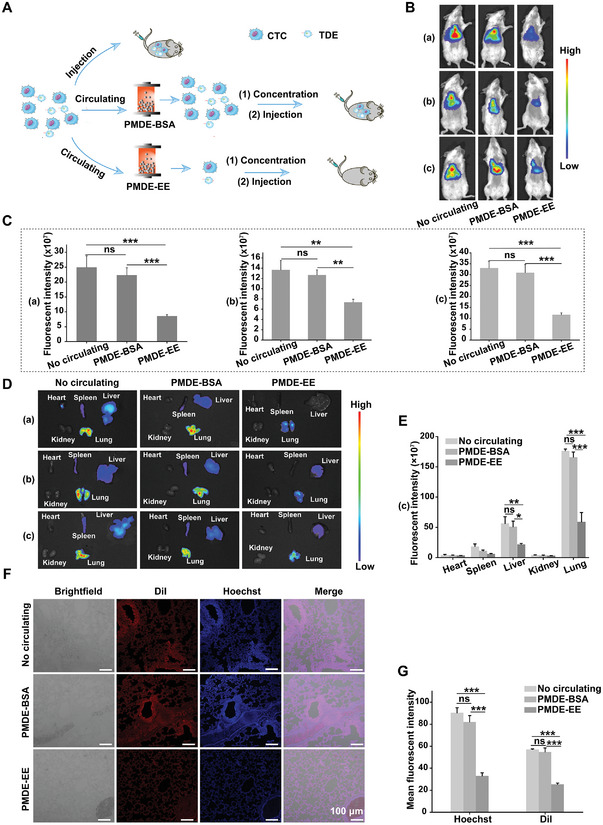
PMDE inhibited the biodistribution of CTCs and TDEs to distant organs. A) Schematic diagram to validate the effect of PMDE on the biodistribution of CTCs and TDEs. B,C) Representative in vivo fluorescence imaging of Balb/c mice at 3 h following injection and its ROS analysis (*n* = 3). (a) Effect of PMDE on the biodistribution of cancer cells; (b) Effect of PMDE on the biodistribution of TDEs; (c) Effect of PMDE on the biodistribution of cancer cells and TDEs. D,E) Representative in vitro fluorescence imaging of Balb/c mice at 3 h following injection and ROS analysis in (c) (*n* = 3). F,G) Representative confocal imaging of the biodistribution of cancer cells and TDEs in the lung of Balb/c mice and its semi‐quantitative analysis (*n* = 3). Scale bar: 100 µm. Data were shown as mean ± SD. *p <0.05, **p <0.01, ***p <0.001 and ns: not significant.

Both CTCs and TDEs are the culprits in the initiation of metastasis. To further investigate the potential of using this PMDE for anti‐metastatic therapies, we conducted a proof‐of‐concept experiment as shown in **Figure** **8**A. To reduce experimental bias, PMDE‐BSA was used as a negative control and the untreated group was used as a blank control. Representative in vivo fluorescence images of mice acquired at 3 h after tail vein injection are displayed in Figure 8B. The fluorescence signals of PMDE‐EE were significantly the lowest regardless of whether the experimental subject was CTCs, TDEs, or both (Figure 8C). Representative in vitro fluorescence images of the heart, liver, spleen, lung, and kidney from mice acquired at 3 h after tail vein injection are showed in Figure 8D. Cancer cells and TDEs were mainly distributed in the lung and liver. Importantly, compared to PMDE‐BSA, PMDE‐EE significantly reduced the biodistribution of cancer cells and TDEs in the lung, and liver (Figure 8E; Figure S23 Supporting Information). These results showed that most of the CTCs and TDEs had been removed by PMDE‐EE before they arrived at distant organs. To further verify the inhibitory effect of PMDE‐EE on the biodistribution of cancer cells and TDEs, MDA‐MB‐468 cells stained with Hoechst and MDA‐MB‐468 cell‐derived exosomes stained with DiI were used. Fluorescence images of lung sections (Figure 8F; Figure S24A,C, Supporting Information) and semiquantification by image J (Figure 8G; Figure S24B,D, Supporting Information) showed that the PMDE‐EE treatment group had the lowest fluorescence intensity regardless of whether the experimental subject was CTCs, TDEs, or both. In addition, fluorescence images of liver sections (Figure S25A,C,E, Supporting Information) and semiquantification by image J (Figure S25B,D,F, Supporting Information) showed the same results. This further indicated that PMDE‐EE simultaneously removed cancer cells and TDEs before they arrived at distant organs. Compared with all the currently available methods for the in vivo removal of CTCs from peripheral blood circulation, this work validated for the first time that the dual‐removal of CTCs and TDEs from the peripheral circulation can inhibit their biodistribution to distant organs, which is certainly promising for anti‐metastatic treatment (Table S2, Supporting Information). In hematogenous metastasis, both CTCs and TDEs are distributed via the blood circulation to distant organs and play an irreplaceable role in the metastatic cascade. TDEs act as messengers sent into the blood circulation by the primary tumor and reach specific distant organs to prepare pre‐metastatic niche for CTC colonization even before their arrival. Importantly, this device can efficiently simultaneously remove CTCs and TDEs from the peripheral blood circulation to impede their arrival at distant organs. This PMDE is expected to prevent pre‐metastatic niche formation and the colonization of CTCs, and its application in anti‐metastatic therapies is promising.

## 3 Conclusion

In the present study, by integrating SMs‐EE with hemoperfusion technology, we developed a pro‐metastatic derivative eliminator (PMDE). The PMDE with excellent biocompatibility was used for the dual‐removal of CTCs and TDEs in the bloodstream to impede their biodistribution in vital distant organs and SMs‐EE were retrieved to collect tumor samples from whole‐body blood for downstream analysis. SMs were immobilized with bivalent tumor‐specific antibodies to enhance the capture efficiency of CTCs and TDEs. Vascular cannulation of the abdominal aorta and inferior vena cava was performed to construct an in vivo circulation model in rats. Importantly, the biodistribution of CTCs and TDEs in vital distant organs was significantly impeded by the efficient removal of CTCs and TDEs from peripheral blood circulation. This PMDE is expected to impede pre‐metastatic niche formation and the colonization of CTCs. In addition, the efficiency of CTCs and TDEs release from SMs‐EE was 92.7 ± 1.2% and 85.6 ± 1.3%, respectively. The released tumor cells maintained excellent cell viability and proliferation capacity, and the TDEs had an intact vesicular structure, indicating that the capture and release had little effect on the tumor cells and the TDEs. It is thus feasible for PMDE to provide tumor samples for downstream analysis by efficiently releasing the captured CTCs and TDEs. The advantages of the PMDE from this work over our previous work are as follow. First, PMDE has a larger specific surface area and can capture tumor exosomes with sizes up to 100 times smaller than that of tumor cells. Second, the capture efficiency of the PMDE is significant enhanced due to dual targets. Finally, PMDE significantly inhibited CTCs and TDEs from reaching distal organs, whereas the whole blood purifier (WBP) did not.^[^ ^11c^ ^]^

In current detection technologies, the volume of blood samples taken for detection is only one‐thousandth of that of the peripheral blood. However, CTCs are rare, and therefore, there is a sampling bias that leads to false negatives. Notably, the PMDE can enrich all CTCs and TDEs as much as possible by contacting the entire peripheral blood supply, thus obtaining a representative sample for tumor diagnosis and detection.

The antibody coupling route used in this study can couple protein‐like molecules with SMs‐Epo, such as anti‐EpCAM antibody, anti‐EGFR antibody, and BSA under the same reaction conditions. Perhaps, in clinical practice, the best identifying markers can be coupled with SMs‐Epo to recognize CTCs and TDEs that are selected according to the cellular phenotype of the tumor patient to realize precise individualized treatment. Additionally, for tumor patients with increased CTCs and TDEs in the bloodstream, regular treatment with PMDE is administered first to reduce the burden of CTCs and TDEs and inhibit their distribution to vital distant organs, thereby mitigating tumor recurrence. Moreover, at the end of each treatment, the individualized immunosorbent in the capture column is retrieved and the captured CTCs and TDEs are released for tumor diagnosis and detection, which helps to analyze the treatment effect as well as to make the next treatment plan. In addition, the biocompatibility tests were carried out to confirm the outstanding hemocompatibility and biocompatibility of this device. Hemoperfusion technology has been applied in the clinic for decades with abundant clinical cases and experiences. Thus, the PMDE will be a promising method to lower the CTC and TDE burden in tumor patients and has great potential for clinical application.

In a follow‐up study, we will construct a patient‐derived tumor xenograft model in the rats to evaluate the anti‐metastatic therapeutic effect of PMDE. In addition to CTCs and TDEs, this device will be used to remove other pro‐metastatic derivatives such as tumor‐induced immunosuppressive cytokines and cells.^[^ ^6^ ^]^ It has been shown that immune surveillance against CTCs can be enhanced by antibody drugs to improve the ability of immune cells to kill and eliminate CTCs.^[^ ^25^ ^]^ Therefore, the combination of drugs and medical devices is promising for anti‐metastatic therapy in the future. In conclusion, the present study provides a promising method for simultaneously reducing the burden of CTCs and TDEs in the bloodstream to prevent their biodistribution in distant organs and has broad prospects in clinical applications to prevent tumor metastasis and recurrence.

## Conflict of Interest

The authors declare no conflict of interest.

## Supporting information

Supporting Information

Supplemental Movie 1

Supplemental Movie 2

## Data Availability

The data that support the findings of this study are available from the corresponding author upon reasonable request.
