# Supplementary material for: A Pro‐Metastatic Derivatives Eliminator for In Vivo Dual‐Removal of Circulating Tumor Cells and Tumor‐Derived Exosomes Impedes their Biodistribution into Distant Organs
Source: Adv Sci (Weinh). 2023 Oct 22;10(34):2304287. doi: 10.1002/advs.202304287 (PMC10700241; doi:10.1002/advs.202304287)
Supplement: Supplementary file 1 — Supporting Information [file ADVS-10-2304287-s003.pdf]

ADVANCED SCIENCE
Open Access

Supporting Information

for *Adv. Sci.*, DOI 10.1002/advs.202304287

A Pro-Metastatic Derivatives Eliminator for In Vivo Dual-Removal of Circulating Tumor Cells and Tumor-Derived Exosomes Impedes their Biodistribution into Distant Organs

*Ying Sun, Lei Xing, Jun Luo, Ming-Tao Yu, Xiao-Jie Wang, Yi Wang, Tian-Jiao Zhou and Hu-Lin Jiang**

Supporting Information

A Pro-Metastatic Derivatives Eliminator for in Vivo Dual-Removal of Circulating Tumor Cells and Tumor-Derived Exosomes Impedes their Biodistribution into Distant Organs

Ying Sun, Lei Xing, Jun Luo, Ming-Tao Yu, Xiao-Jie Wang, Yi Wang, Tian-Jiao Zhou, Hu-Lin Jiang *

Experimental Section

Materials: Sepharose 6FF was purchased from Beijing RuiDaHengHui Science&Technology Development Co., Ltd.. (Beijing, China). Epichlorohydrin was purchased from Aladdin Chemistry (Shanghai, China). Anti-EpCAM antibody, anti-EGFR antibody, Alexa Fluor® 647-labeled anti-EpCAM antibody, FITC-labeled anti-EGFR antibody, FITC-labeled anti-EpCAM antibody, anti-CD63 antibody, and TSG101 antibody were purchased from Santa Cruz Biotechnology, Inc. (Santa Cruz, CA, USA). FITC-BSA was purchased from Solarbio (Beijing, China). Anti-CD45 antibody was purchased from Invitrogen (Waltham, USA). Human serum albumin was purchased from Aladdin (Shanghai, China). FITC-HSA was purchased from Beijing Bersee Science and Technology Co., Ltd.. (Beijing, China). Alexa Fluor® 647-labeled goat anti-mouse IgG, Alexa Fluor® 647-labeled goat anti-rabbit IgG, dyeing solution (DAPI, DiD, Hoechst 33342 and DiI), CD81 rabbit monoclonal antibody, Calcein/PI assay kit, BCA protein assay kit, and HRP-conjugated goat anti-mouse IgG were purchased from Beyotime Biotechnology (Shanghai, China). DMEM medium, CCK-8 kit, and Erythrocyte lysis solution were purchased from

KeyGEN BioTECH (Nanjing, China). Trypsin-EDTA and Fetal bovine serum (FBS) were purchased from Gibco Life Technologies. Opti-MEM media were purchased from Thermo Fisher Scientific (Waltham, USA). DiR was purchased from Baisai Biochemicals (Shanghai, China). Special lysate for exosomal proteins (UR33101) was obtained from UmibioScience and Technology. Rat Platelet Factor 4 (PF_4) kit was obtained from Mlbio Biotechnology Co., Ltd..(Shanghai, China). Sterile single-use syringes were purchased from Jiangsu ChangCheng Medical Equipment Co., Ltd.. Indwelling needles were purchased from Zhejiang Kangdeli Medical Equipment Co., Ltd..

Preparation of SMs-Epo: To activate sepharose microspheres (SMs) with epichlorohydrin, 300 mg of washed and suction-dried Sepharose 6FF were suspended in 3.5 mL of dimethyl sulfoxide (DMSO) and mixed with 1 mL of 0.4 M sodium hydroxide solution, then 0.5 mL of epichlorohydrin was added. The suspension was incubated at room temperature (RT) for 5 h with shaking,100 rpm (THZ-312, JingHong, Shanghai, China). It was then transferred to a glass filter funnel and SMs was washed with 100 mL of ultrapure water.

Measurement of the epoxy density: SMs-Epo was sampled to measure the epoxy density grafted on the SMs according to the method of Sundberg and Porath[1] and expressed as micromole per gram of suction-dried SMs-Epo. In brief, The washed SMs-Epo was filtrated to obtain the filter cake. Then, 200 mg SMs-Epo was mixed with 3 mL of $Na_2S_2O_3$ (1.3 M in ultrapure water) and 100 μL phenolphthalein and stirred at RT for 30 min. The mixture was titrated with hydrochloric acid standard

solution (0.1 M in ultrapure water) until the color faded. The density of epoxy was calculated by Equation (1).

$$D_{epoxy} = M_{HCl} \cdot \frac{V_{HCl}}{M_g} \qquad (1)$$

in which M_{HCl} was the concentration of the hydrochloric acid standard solution (0.1 M). V_{HCl} was the volume of hydrochloric acid standard solution used to neutralize the mixture. M_g was the weight of the suction-dried SMs-Epo.

Preparation and characterization of immunosorbents: Firstly, antibody solutions were prepared in carbonate buffer (0.1M, pH=11): a. 500 μL of 20 μg mL^{-1} anti-EpCAM antibody; b. 500 μL of 20 μg mL^{-1} anti-EGFR antibody; c. 500 μL of 20 μg mL^{-1} anti-EpCAM antibody and anti-EGFR antibody; d. 500 μL of 20 μg mL^{-1} BSA. Then, each of the above four solutions was added to a 2 mL Eppendorf (EP) tube containing 50 mg SMs-Epo and was placed in a constant temperature shaker at 37°C, 100 rpm, overnight. To visualize the antibody-functionalized SMs, coupling experiments were performed using a FITC-labeled anti-EGFR antibody, Alexa Fluor® 647-labeled anti-EpCAM antibody, and FITC-BSA. Briefly, These SMs were washed three times with PBS, imaged, and photographed using an inverted fluorescence microscope (Nikon Eclipse Ti., Nikon, Japan) or confocal laser scanning microscopy (CLSM) (LSM700, Carl Zeiss, Germany), and the average fluorescence intensity was calculated using Image J software. To further characterize antibodies immobilized on SMs successfully, Alexa Fluor® 647-labeled goat anti-mouse IgG was used to specifically bind mouse-derived anti-EpCAM antibody and anti-EGFR antibody coupled on the surface of the microspheres. Briefly, SMs-EpCAM and SMs-EGFR

were washed three times with PBS, and 500 μL of Alexa Fluor® 647-labeled goat anti-mouse IgG (1:100 PBS dilution) was incubated with SMs-EpCAM and SMs-EGFR for 3 h, at 37°C, respectively. Then, SMs-EpCAM and SMs-EGFR were washed three times with PBS, imaged, and photographed using CLSM.

Measurement of the antibody coupling density and efficiency: To further characterize the coupling density and the coupling efficiency, a standard curve of fluorophore-labeled antibody was first prepared with fluorescence intensity as the vertical coordinate and antibody concentrations as the horizontal coordinate. The coupling density (C_E) and coupling efficiency (C_D) was calculated according to Equation (2) and Equation (3), respectively.

$$C_D = \frac{(C_1 - C_2)\cdot V}{M_g}\,(\mu g/mg) \quad or \quad C_D = \frac{n\cdot NA}{M_g}\,(binding\ sites/mg) \tag{2}$$

$$C_E = \frac{C_1 - C_2}{C_1} \tag{3}$$

Here, C_1 (μg mL^{-1}) is the initial concentration of fluorescent antibodies; C_2 (μg mL^{-1}) is the residual concentration of fluorescent antibodies, which is calculated according to the standard curve; V (mL) is the volume of fluorescent antibody; Mg (mg) is the mass of the SMs; n (mol) is the number of moles of coupled antibody; NA is Avogadro constant. The fluorescence intensity of fluorescent antibodies was determined using a Multifunctional Enzyme Analyzer (Molecular Devices, USA).

Cell culture: MDA-MB-468, MDA-MB-231, HeLa, L02, and HUVEC cells were purchased from the Chinese Academy of Sciences Shanghai Institute of Cell Bank (Shanghai, China) and cultured in Dulbecco's Modified Eagle medium (DMEM) supplemented with 10% fetal bovine serum (FBS) and 1% antibiotics

(penicillin-streptomycin) at 37°C in a humidified incubator containing 5% CO_2.

EpCAM and EFGR expression in MDA-MB-468 and HeLa cells: EpCAM and EFGR expression in MDA-MB-468 and HeLa cells were analyzed by immunofluorescence. The cells were fixed with 4% PFA for 20 min at RT and washed with PBS. Fixed cells were washed three times with PBS and then blocked with 5% BSA/PBS for 30 min at 37°C. Then cells were incubated with EpCAM mAb (1:100 dilution) or EGFR mAb (1:100 dilution) overnight at 4°C. After washing three times with PBS, the cells were incubated with Alexa Fluor® 647-labeled goat anti-mouse IgG (1:500 dilution) for 1 h in the dark at 37°C. Then, cell nuclei were stained with DAPI in the dark at RT for 5 min after washing three times with PBS. The cells were washed three times with PBS and the images were visualized by CLSM.

Antibody activity examination: anti-EpCAM and anti-EGFR antibody were incubated in coupling buffer (carbonate buffer) for 12h at 37°C. This antibody was used for immunofluorescence experiments of MDA-MB-468 cell surface markers EpCAM and EGFR according to the above method. Untreated antibody was used as a control group for imaging with CLSM.

Preparation of model exosomes: Model exosomes were obtained from MDA-MB-468 cells culture medium by differential ultracentrifugation, according to the protocol described by Th´ery et al[2] with some modifications. In brief, once 80% cell confluency was achieved, the media was carefully removed and the cells were washed twice with PBS. Then Opti-MEM media was added to the culture dish to replace the DMEM media and cultured for another 48 h. The media was collected to prepare

model exosomes by ultracentrifugation. The collected medium was centrifuged at 300 g for 20 min, 2000 g for 20 min, and then 10000 g for 30 min to remove cells, dead cells, and cell debris, respectively. (Centrifuge 5810R, Eppendorf, Germany). Afterward, the supernatants were ultracentrifuged at 110 000 g (SW 32 Ti rotor, Beckman Coulter, USA) for 70 min at 4°C to pellet the crude exosomes. The pellets were washed twice with PBS, resuspended in a defined amount of PBS, and stored at -80°C.

The protein quantification of exosomes: BCA kit was utilized to measure the protein quantification of exosomes according to the reagent instructions. In brief, the BCA working solution was configured according to the 50:1 ratio of reagent A: reagent B in the BCA kit. Then, 200 μL of BCA working solution were added to each well in the 96 well plate, and 20 μL of the extracted protein sample were added to 96-well plate. Next, the plate was incubated at 37 °C for 30 min. A microplate reader was used to determine the absorbance of the sample at 562 nm. A standard curve was drawn, and the protein content in the sample solution was calculated according to the standard curve.

Static capture and characterization of CTCs: Firstly,50 mg of SMs-EpCAM was taken in a 2 mL EP tube, then added 2×10^5 MDA-MB-468 cells and supplemented PBS to 500 μL, and then placed in a constant temperature shaker at 37°C, 100 rpm, for 1 h. To enable visualization of SMs-EpCAM that had captured CTC, a FITC-labeled anti-EpCAM antibody was used as a capture antibody, while tumor cells were labeled with DiD. After captured, SMs-EpCAM was washed three times

with PBS to remove non-specific molecules adsorbed on the surface of the microspheres, stereoscopically (Overlay z-stock mode, taking one picture every 5 μm) imaged and photographed using CLSM.

Static capture and characterization of TDEs: First, 50 mg of SMs-EpCAM was placed in a 2 mL EP tube, and then added 50 μg (total exosome protein) of MDA-MB-468 cells derived exosomes and supplemented PBS to 500 μL, then were shaken at a constant temperature of 37°C, 100 rpm, for 1h. To enable the visualization of SMs-EpCAM that had captured TDEs, immunofluorescence staining analysis was used for characterization. After centrifugation (1000 rpm, 3 min), the supernatant was discarded and SMs-EpCAM was thoroughly washed three times with PBS to remove non-specific molecules adsorbed on the surface of the microspheres. To verify the specific adsorption of exosomes on SMs-EpCAM, three isotype controls (SMs-EpCAM+cell lysate; SMs-EpCAM+exosome lysate; SMs-BSA+cell lysate) were set up and samples were blocked at room temperature for 1 h in 5% BSA/PBS and then incubated with CD81 rabbit monoclonal antibody (1:200 dilution) at 4 °C overnight. Subsequently, the samples were stained with Alexa Fluor® 647-labeled goat anti-rabbit IgG (1:500 dilution) for 1 h at RT. Finally, CLSM was used to image.

Establishment of calculation methods for capture efficiency: MDA-MB-468 cells were stained with DAPI for 10 min at RT in the dark and a series of cell density gradients were set. Fluorescence spectra of different density gradients of MDA-MB-468 cells were recorded using a Multifunctional Enzyme Analyzer, with emission slit set at 5 nm, and excitation set at 360 nm. Fluorescence emission was

exhibited in the 400-560 nm field. Meanwhile, the fluorescence intensity at the excitation wavelength of 360 nm and emission wavelength of 460 nm was recorded for fitting the standard curve. Exosomes were pre-stained with DiR, and Amicon Ultra 100-kDa filters (Millipore Sigma) were used to wash away the excess DiR. Fluorescence spectras of exosomes were recorded using a Multifunctional Enzyme Analyzer, with emission slit set at 10 nm, and excitation set at 708 nm. Fluorescence emission was exhibited in the 748–818 nm field. Also, the fluorescence intensity with excitation wavelength at 708 nm and emission wavelength at 768 nm was recorded for fitting the standard curve. The capture efficiency of CTCs and TDEs was calculated according to Equation (4) and Equation (5), respectively.

$$C_E = \frac{N_1 - N_2}{N_1} \tag{4}$$

$$C_E = \frac{M_1 - M_2}{M_1} \tag{5}$$

where C_E refers to the capture efficiency of CTCs or TDEs, N_1 refers to the initial count of CTCs added, and N_2 refers to the count of dissociative CTCs in the supernatant after capture, which is calculated based on the fluorescence intensity of the sample, the volume, and the standard curve. M_1 refers to the initial amount (μg) of TDEs added, and M_2 refers to the amount of dissociative TDEs in the supernatant after capture, which is calculated based on the fluorescence intensity of the sample, the volume, and the standard curve. The fluorescence intensity was measured using a Multifunctional Enzyme Analyzer.

In vitro circulating simulation experiments: Since both CTCs and TDEs are present in the bloodstream of cancer patients, we added MDA-MB-468 cells and MDA-MB-468

cells derived exosomes simultaneously to perform in vitro circulating simulation experiments and investigate the factors affecting the capture efficiency. Firstly, the PMDE device was installed. Briefly, the PMDE consists of intravenous catheters (ABLE®, Guangdong, China), a capture column, and a peristaltic pump (F01A-STP, Kamoer, Shanghai, China) connected in sequence. The intravenous catheter is made of silicone and its inner and outer diameters are 6.4 mm, and 9.6 mm respectively. The capture column was modified from a 2.5 mL syringe. Briefly, the piston of the 2.5 mL syringe is withdrawn, a 300 mesh screen is laid on the bottom of the column, which is then filled with 300 mg of SMs-EE, and finally, the piston is added, which is connected to an intravenous catheter through a modified syringe needle. Then 10 mL PBS with 2×10^5 MDA-MB-468 cells stained with DAPI in advance (total volume 200 μL in PBS) and 50 μg MDA-MB-468 cells-derived exosomes labeled with DiR in advance (total volume 200 μL in PBS,) were added to the ampoule bottle; the peristaltic pump () was turned on, adjusted to the specified flow rate, cycled for the specified time, then the circulating medium was collected to separate the uncaptured MDA-MB-468 cells and MDA-MB-468 cells-derived exosomes. Specifically, the circulating medium was centrifuged at 1000 rpm for 3 min; the supernatant was concentrated to 200 μL with an Amicon Ultra 10-kDa filter (Millipore Sigma) and cell precipitation was resuspended with 200 μL PBS. Similarly, we used Equation (4) and Equation (5) to calculate the capture efficiency of CTCs and TDEs, respectively. To examine the effect of packing volume in the capture column on the capture efficiency of CTCs and TDEs. The amount of 100, 200, 300, 400, and 500 mg

SMs-EE in the capture column was set respectively and was carried out in vitro circulating simulation experiments according to the above method and calculated the capture efficiency. The effect of circulating time on the capture efficiency of CTCs and TDEs was investigated. The groups of 10, 20, 30, 40, 50, and 60 min were set up respectively, and the in vitro circulating simulation experiments were performed according to the above method, and the capture efficiency was calculated. To investigate the effect of circulating flow rate on the capture efficiency of CTCs and TDEs, the groups of 5, 10, 15, and 20 mL min^{-1} were set up respectively, and the in vitro circulating simulation experiments were performed according to the above method, and the capture efficiency was calculated. To examine the specificity of SMs-EE to capture CTCs and TDEs, the high-EpCAM group (MDA-MB-468 cells and their exosomes), low-EpCAM group (MDA-MB-231 cells and their exosomes), and negative control (HeLa cells and HeLa cells derived exosomes) were set up, respectively. The in vitro circulating simulation experiments were performed according to the above method, and the capture efficiency was calculated. To examine the effect of circulating medium on the capture efficiency, we used whole blood (Blood was collected from the abdominal aorta of rats), platelet-poor plasma (PPP), and PBS as a circulating medium and performed in vitro circulating simulation experiments according to the above method and calculated the capture efficiency. The effect of the interaction between CTCs and TDEs on the capture efficiency was investigated. First, 50 μg of MDA-MB-468 cells-derived exosomes were fixed, and the count of MDA-MB-468 cells was increased sequentially from 2×10^5 to 10^6

MDA-MB-468 cells, and the in vitro circulating experiments were performed as described above. Then, MDA-MB-468 cells were fixed for 2×10^5, thereby increasing the amount of exosomes, from 50 μg to 90 μg. The in vitro circulating simulation experiments were performed according to the above method, and the capture efficiency was calculated.

Release of CTCs and TDEs from SMs-EE: After circulating, SMs-EE was retrieved from the capture column, first incubated with 0.25 % trypsin-EDTA for 3 min at RT, followed by dilution with PBS, filtered through a 300 mesh sieve, and then the cell precipitate was collected from the filtrate at 2000 rpm, 3 min and then used for reculture. The supernatant was reserved and named Supernatant 1. The SMs-EE was then incubated with pH 2.2 glycine-HCl buffer for 5 min at RT, and the supernatant was collected at 1000 rpm for 3 min and named Supernatant 2. Finally, Supernatant 1 and Supernatant 2 were mixed and concentrated with Amicon Ultra 10-kDa filters (Millipore Sigma) for Western blot assay, NTA assay, and TEM photography. The release efficiency of CTCs and TDEs was calculated according to Equation (6) and Equation (7) below.

$$R_E = \frac{\mathrm{N}_3}{N_1 - N_2} \quad (6)$$

$$R_E = \frac{\mathrm{M}_3}{M_1 - M_2} \quad (7)$$

Here, where R_E refers to the release efficiency of CTCs or TDEs, N_1 refers to the initial count of CTCs added, and N_2 refers to the count of dissociative CTCs in the supernatant after capture, which is calculated based on the fluorescence intensity of the sample, the volume, and the standard curve. N_3 refers to the count of released

CTCs in PBS, which is calculated based on the fluorescence intensity of the sample, the volume, and the standard curve. M_1 refers to the initial amount (μg) of TDEs added, and M_2 refers to the amount of dissociative TDEs in the supernatant after capture, which is calculated based on the fluorescence intensity of the sample, the volume, and the standard curve. M_3 refers to the amount of released TDEs in PBS, which is calculated based on the fluorescence intensity of the sample, the volume, and the standard curve. The fluorescence intensity was measured using a Multifunctional Enzyme Analyzer.

Calcein-AM / PI double-staining assay: The released cells were co-stained using Calcein-AM and PI for 15min, washed three times using PBS, added on a slide, covered with a coverslip, and observed under an inverted fluorescence microscope for photographs.

Cell viability: The released cells were inoculated into 96-well plates with 100 μL DMEM (supplemented with 10% FBS) per well and cultured for 5 days. On days 1, 3, and 5, respectively, cell viability was measured using the CCK-8 kit, and OD values at 450 nm were recorded using a Microplate Reader (Thermo, USA).

Western blot assay of MDA-MB-468 cells-derived exosomes: Exosomes from ultracentrifugation or SMs-EE were lysed with special lysate for exosomal proteins. Special lysate for exosomal proteins (UR33101, UmibioScience and Technology) was used to lyse exosomes according to the reagent instructions. In brief, the exosome samples were mixed with the lysate at a volume ratio of 1:1 and lysed on ice for 10 min, then centrifuged at 12,000g for 5 min at 4°C to collect the supernatant. Next, the

concentration of total protein in the supernatant was determined using the BCA protein assay kit. The supernatant was mixed with 5× protein loading buffer and boiled for 5 min to denature the protein. Equal total exosome protein was separated in 10% SDS-PAGE and electrically transferred to a polyvinylidene difluoride (PVDF) membrane. The membrane was blocked with 5 % skim milk in Tris-buffered saline/0.1% Tween-20 (TBST), and incubated with anti-CD63 antibody and TSG101 antibody (diluted at 1:1000) overnight at 4°C. After washing three times with TBST, the membrane was incubated with HRP-conjugated anti-mouse IgG antibody (diluted at 1:1000) at room temperature for 2 h. Finally, it was washed three times with TBST, and 1 mL of chemiluminescence detection reagent was added dropwise, and placed in the imager (Tanon 4200, Shanghai, China) for imaging and taking pictures. The relative purity of exosomes released from SMs-EE was calculated according to Equation (8) below.

$$P_R = \frac{G_{SM-EE}}{G_{UC}}$$

(8)

Here, P_R refers to the relative purity of the exosomes released from SMs-EE compared to those isolated by ultracentrifugation. $G_{SMs\text{-}EE}$ refers to the grayscale value of the protein band generated by exosome maker CD63 when exosomes are from SMs-EE; G_{UC} refers to the grayscale value of protein band generated by exosome maker CD63 when exosomes are from ultracentrifugation. The grayscale value was calculated using Image J software.

Morphology and characterization of the released TDEs by transmission electron

microscopy (TEM): TEM images of exosomes released from SMs-EE were acquired by an H-7800 TEM system (Hitachi, Tokyo, Japan). PBS-diluted samples were stained with 2% phosphomolybdic acid after drying on the Formvar-coated copper grids.

Size and characterization of the released TDEs by nanoparticle tracking analysis (NTA): NTA was carried out by Zetaview (Particle Metrix, Germany). The Zetaview was first calibrated using a 100 nm standard substance. PBS was used to dilute the released exosome samples to evaluate particle size, and the results were displayed in the form of Particles/mL - Diameter (nm).

Hemolysis rate: The hemolysis test was carried out to evaluate the erythrocyte compatibility of SMs-EE. Blood from a rat was collected using vacuum tubes (5 mL, Jiangsu Kangjian Inc., China) containing sodium citrate. Rat whole blood was centrifugated at 2000 rpm for 10 min for isolating red blood cells (RBCs) from plasma and the centrifugation procedure was repeated until the supernatant was clarified and colorless. The separated erythrocyte suspension was configured with saline to 2% (V/V) of erythrocyte suspension. The diluted erythrocyte suspension was added to 50 mg of SMs-EE previously soaked overnight in PBS and incubated for 3 h at 37°C. Saline was chosen as a negative control and deionized water as a positive control. The suspension was then centrifuged at 3000 rpm for 5 min and the absorbance of the hemoglobin released from the suspension was measured at 540 nm using a Microplate Reader, and the hemolysis rate of SMs-EE could then be calculated by Equation(9).

$$Haemolysis\ ratio(\%) = \frac{A_s - A_n}{A_p - A_n} \times 100 \quad (9)$$

Where A_s is the absorbance of the samples, A_p and A_n are the absorbances of the positive control and negative control, respectively.

Protein adsorption: Protein adsorption experiments were carried out with HSA solution under static conditions. Firstly, 50 mg SMs-EE was pre-immersed in PBS overnight and then incubated at 37 °C for 1 h. Then the microspheres were immersed in PBS solution, containing HSA with a concentration of 1 mg mL^{-1}, and incubated at 37 °C for 1 h. The supernatant protein concentration was determined using a BCA kit. Protein adsorption of SMs-EE (μg mg^{-1}) was calculated according to Equation (10).

$$\text{Protein adsorpted } (\mu g/mg) = \frac{(C_1 - C_2)V}{M} \quad (10)$$

Here, C_1 refers to the initial concentration of HSA, C_2 refers to the residual concentration of HSA, V refers to the volume of HSA solution, and M refers to the weight of SMs-EE added. In addition, the distribution of proteins adsorbed on the surface of SMs-EE was explored using FITC-labeled HSA. First, 1 mg L^{-1} of FITC-HSA was dissolved in PBS, and then 50 mg of SMs-EE was immersed in this solution for 1 h at 37°C. Finally, the SMs-EE was washed three times with PBS and observed under an inverted fluorescence microscope.

Evaluation of platelet activation: Platelet activation was evaluated by enzyme-linked immunosorbent assay using the Rat Platelet Factor 4 (PF_4) kit. SMs-EE (10 mg) was pre-soaked in PBS and placed in 1.5 mL EP tubes overnight. Then, PBS was removed, and 150 μL of rat whole blood was added. After incubation at 37°C for 1 h, the whole blood was centrifuged at 2500 g for 10 min (2-8°C) to obtain plasma. Next,

enzyme-linked immunosorbent assays were performed according to the instructions of the Rat Platelet Factor 4 (PF_4) kit.

Evaluation of blood routine: First, 50 mg of SMs-EE was pre-soaked in 1.5 mL EP tubes of PBS (pH = 7.4) overnight and equilibrated at 37°C for 1 h. Secondly, fresh whole blood from rats was collected using EDTA-K2 anticoagulated blood collection tubes. After the removal of PBS, 500 μL of fresh rat whole blood was introduced into each tube. Then, SMs-EE was incubated with whole blood for 60 min at 37°C and then collected the remaining blood. Differential counts of whole blood cells were measured with an automated hematology cell analyzer (BC-2800vet, Mindray Biomedical Electronics Co., Ltd., Shenzhen, China) according to the instructions provided.

Blood coagulation analysis in PPP after incubation with SMs-EE: The procedure was as follows:10 mL of rat blood was added to sodium citrate anticoagulation collection tubes and then centrifuged at 4000 rpm for 15 min to obtain PPP. Then 0.5 mL of PPP was added to 50 mg of SMs-EE in a 1.5 mL EP tube and shaken gently at 37°C for 1 h. PPP without SMs-EE was the control treatment. The tests were performed by an automatic blood coagulation analyzer RAC-1830 (Shenzhen Rayto Life Science Co, China).

Cytotoxicity tests: MDA-MB-468, HeLa, HUVEC, and L02 cells were grown in DMEM supplemented with 10% FBS and 1% antibiotics (penicillin-streptomycin) in a humidified incubator at 37°C with 5% CO2. The cytotoxicity of SMs-EE was assessed by a CCK-8 cell viability test kit. Before the measurement, 2 g of SMs-EE

was pre-soaked in DMEM (10 mL) and incubated at 37°C for 24 h. The supernatant was then filtered and sterilized with a 0.22 μm microporous membrane to obtain the supernatant. Cells at the required density (1×10^4 cells per well) were seeded in 96 well plates for 24 h. After washing with PBS, 100 μL of supernatants was added into the culture medium and incubated at 37°C for 24 h under the above conditions. The control group was incubated with 100 μL culture medium DMEM without SMs-EE and the blank group contains DMEM medium and CCK-8 without cells. Then, CCK-8 solution (10 μL) was added and incubated for 4 h. The OD value was obtained at 450 nm by using a Microplate Reader. Each sample was tested six times in parallel. Cell viability was calculated according to Equation (11).

$$Cell\ viability(\%) = \frac{A_s - A_b}{A_c - A_b} \times 100 \tag{11}$$

Where, A_s refers to the absorbance of experimental wells, A_b refers to the absorbance of blank wells, and A_c refers to the absorbance of control wells.

In vivo circulating experiments in a rat model: All animal experiments were conducted according to the experimental practices and standards approved by the Ethics Committee of China Pharmaceutical University (Approval ID: 2022-10-014). SD rats (male, 280-300 g), 9 weeks old, were kept in pathogen-free conditions for seven days. Experimental procedures were conducted under general anesthesia induced by intramuscular injection of 5% pentobarbital (10 mg kg^{-1}). The surgical procedure was as follows: Firstly, the PMDE device was installed, and the catheter was first filled with sodium heparin solution. Air bubbles were removed from the catheter, and an indwelling needle was attached to the end of the catheter; then the

indwelling needle was inserted into the abdominal aorta and inferior vena cava, and the indwelling needle and vessel were fixed with surgical sutures. At the end of the procedure, the peristaltic pump was turned on (10 mL min^{-1}), and then 2×10^5 MDA-MB-468 cells stained with DAPI and 50 μg of MDA-MB-468 cells-derived exosomes stained with DiR were injected through the tee port of the catheter. The circulating time was 30 min and 1 mL blood samples were collected through the tee port of the catheter at the 1st and 30th minutes for the calculation of capture efficiency. Sodium heparin solution was injected through the tee port of the catheter every 5 min during this period. The blood samples were processed as follows: First, the collected blood samples were centrifuged at 2000 rpm for 3 min. Then, the supernatant was collected and concentrated to 200 μL using an Amicon Ultra 10-kDa filter for calculating of capture efficiency of TDEs. Next, for cell precipitation, erythrocytes were lysed using erythrocyte lysis solution, and then the remaining cells were resuspended with 200 μL PBS for calculating of capture efficiency of cancer cells. Finally, The fluorescence intensity was measured using a Multifunctional Enzyme Analyzer. Similarly, we used Equation (12) to calculate the capture efficiency.

$$C_E = \frac{F_1 - F_2}{F_1} \tag{12}$$

Where F_1 refers to the fluorescence intensity generated by CTCs or TDEs in 1ml blood at the 1st minute of circulating; F_2 refers to the fluorescence intensity generated by CTCs or TDEs in 1ml blood at the 30th minute of circulating.

In vivo toxicity of PMDE: To investigate the in vivo toxicity of PMDE, blood samples were collected from the tail vein on days 1, 7, and 14 for routine blood tests and blood

biochemical analysis by automated hematology cell analyzer and automatic biochemical analyzer (Chemray 240, Mindray Biomedical Electronics Co., Ltd., Shenzhen, China). Rat weight was monitored every two days after surgery. The rats were sacrificed on day 14, and then, major organs, including the heart, liver, spleen, lung, and kidney, were collected and stained with hematoxylin and eosin (H&E).

In vivo and ex vivo fluorescence imaging: First, 2×10^5 MDA-MB-468 cells and 50 μg MDA-MB-468 cells-derived exosomes were stained with DiR, respectively, then was performed in vitro circulating for 30min at 10mL min^{-1}. To reduce the bias brought by the experimental procedure, the PMDE-BSA group was used as a negative control and the untreated group as a positive control. i: CTCs as the experimental subject: after circulating, the circulating medium was centrifuged for 2000 rpm, 3 min, and the precipitate was resuspended with 100 μL PBS, injected into the tail vein of Balb/c mice, and after an interval of 3 h, in vivo, fluorescence imaging was performed (IVIS® spectrum, PerkinElmer, USA). ii: TDEs as experimental subjects: the circulating medium was concentrated to 100μL using Amicon Ultra 10-kDa filters, injected into the tail vein of Balb/c mice, and after an interval of 3 h, in vivo fluorescence imaging was performed. iii: CTCs and TDEs as experimental subjects: The circulating medium was centrifuged at 2000 rpm for 3 min. The precipitate was resuspended with 100 μL PBS; the supernatant was concentrated to 100 μL using an Amicon Ultra 10-kDa filter. Then the above-concentrated solution was injected into the tail vein of Balb/c mice, and after 3h intervals, in vivo fluorescence imaging was performed. After all, in vivo fluorescence imaging was completed, and the heart, liver,

spleen, lung, and kidney of each mouse were collected for in vitro fluorescence imaging.

Tissue section scanning: MDA-MB-468 cells were stained with Hoechst, and exosomes were stained with DiI. Operating as described above, 3 h after tail vein injection, the liver and lung of each mouse were collected for paraffin sectioning, followed by imaging using CLSM, and the average fluorescence intensity was calculated using Image J software (National Institutes of Health, USA).

Statistical Analysis: All data were shown as means ± standard deviations (SD). To compare significant differences among the experimental data, unpaired two-tailed t-tests were used for two-group comparisons and the one-way analysis of variance (ANOVA) with LSD was used for multiple comparisons. All tests were two-sided, the $p > 0.05$ presented not significant (ns), $*p < 0.05$ presented significant, and $**p < 0.01$ even $***p < 0.001$ presented highly significant. Comparisons of all groups were analyzed using the SPSS 19.0.

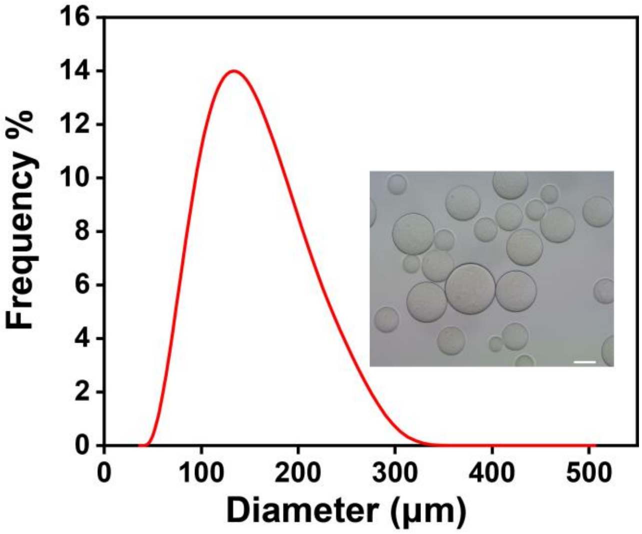

Figure S1. The size distribution of sepharose microspheres. Scale bar: 100 μm.

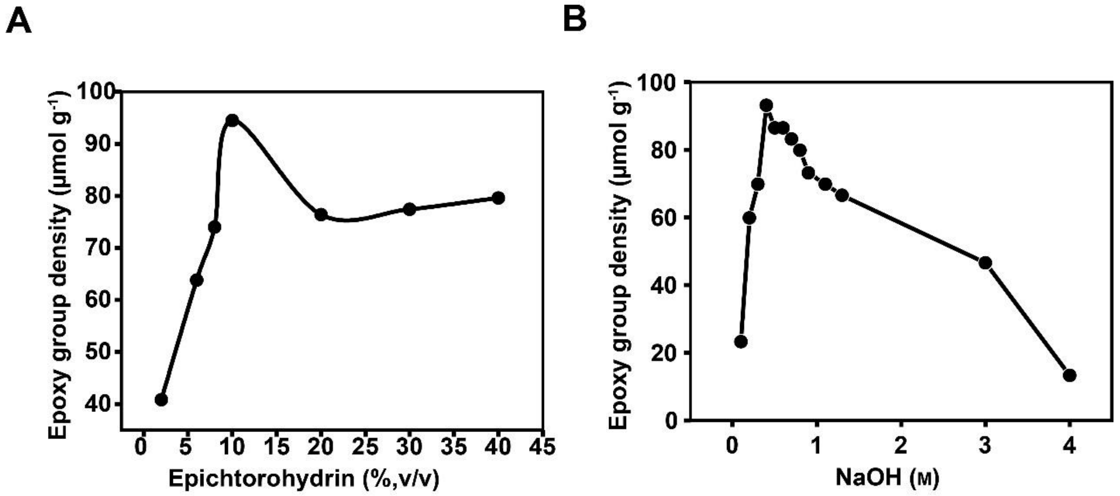

Figure S2. The optimal concentration of A) ECH and B) NaOH reacting with SMs.

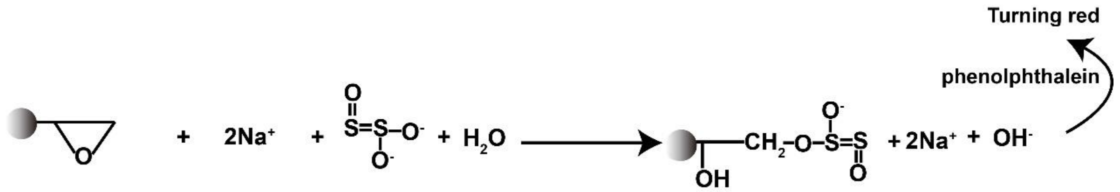

Figure S3. Principle of color development of SMs-Epo in sodium thiosulfate-phenolphthalein solution.

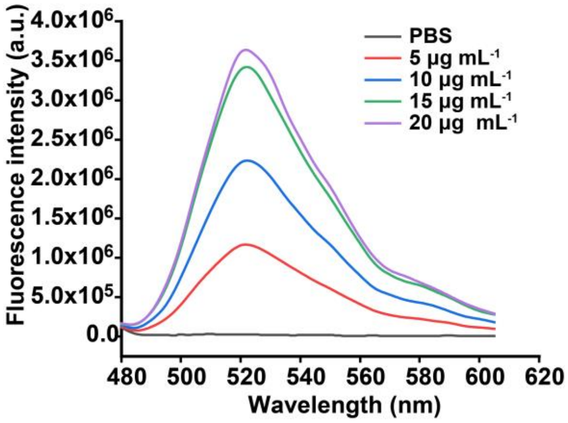

Figure S4. Fluorescence spectra of different concentrations of FITC-labeled anti-EpCAM antibody.

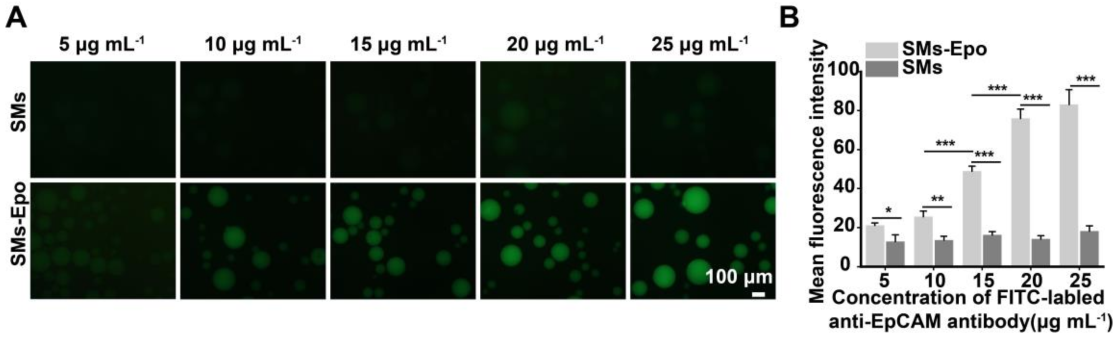

Figure S5. A) Fluorescence images of SMs-Epo incubated with different concentrations of FITC-labeled anti-EpCAM antibody from 5 to 25 μg mL^{-1}. Scale bar: 100 μm. B) Quantitative analysis of the fluorescence intensity using Image J software (n=3). Data are the mean ± SD. *p <0.05, **p <0.01, ***p <0.001.

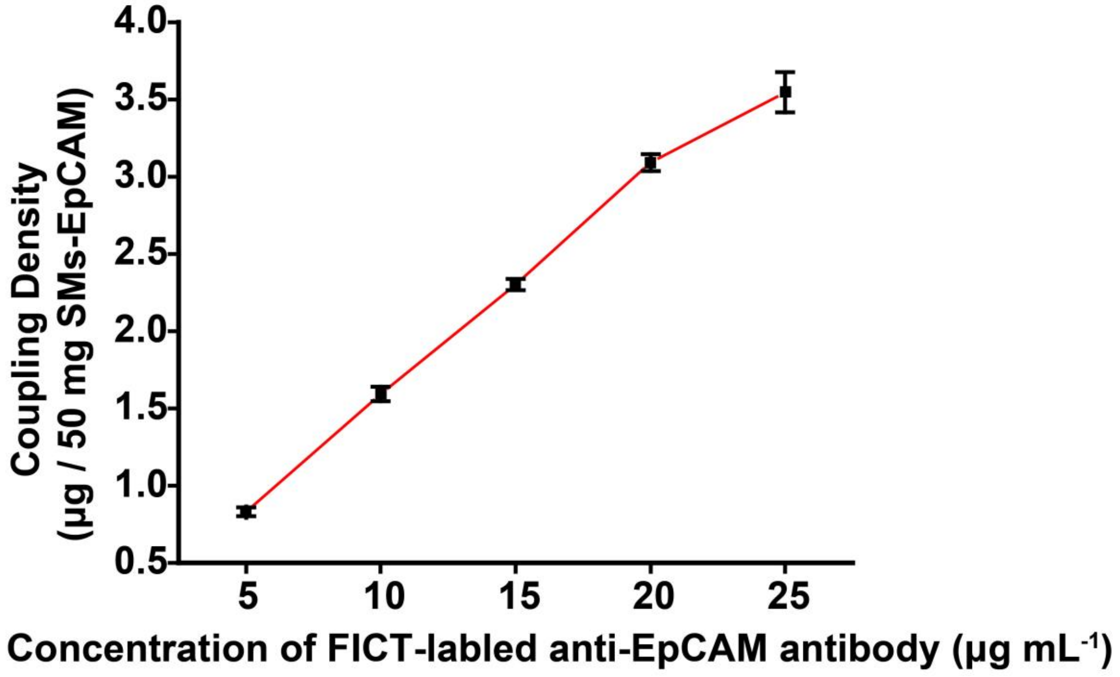

Figure S6. The coupling density of anti-EpCAM antibody at different concentrations (n=3). Data are the mean ± SD.

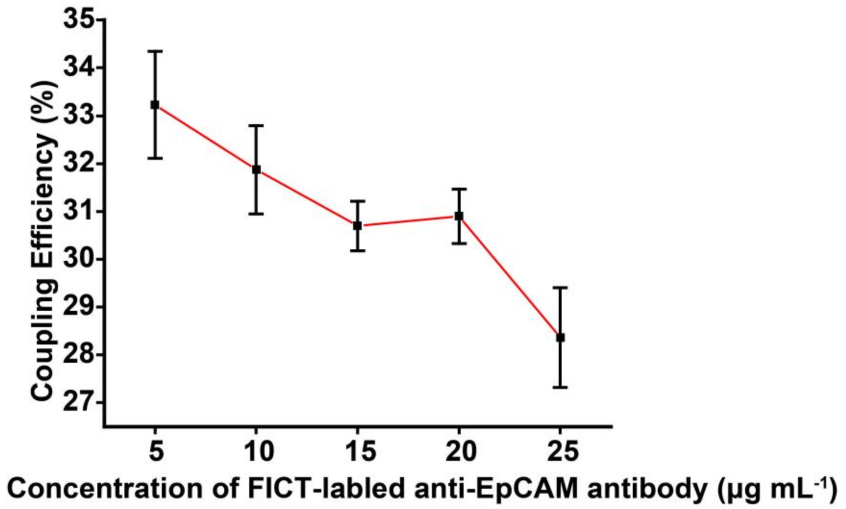

Figure S7. The coupling efficiency of anti-EpCAM antibody at different concentrations (n=3). Data are the mean ± SD.

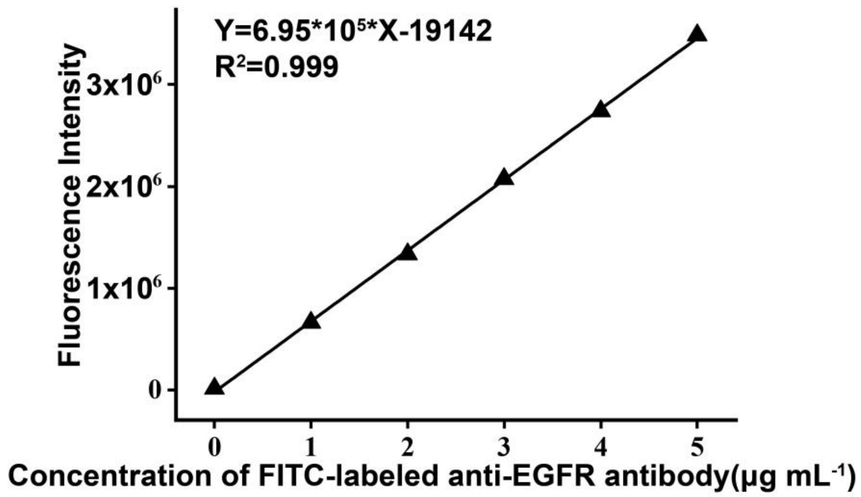

Figure S8. The linear relationship between the concentrations of FITC-labeled anti-EGFR antibody and fluorescence intensity.

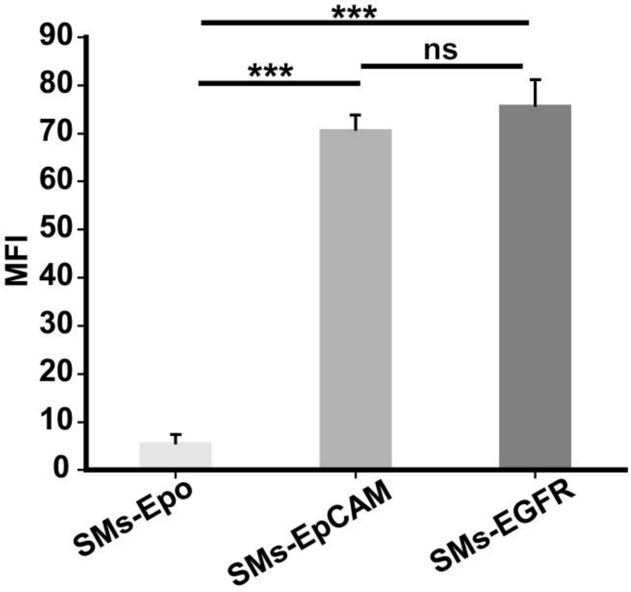

Figure S9. SMs-Epo was incubated with anti-EpCAM antibody and anti-EGFR antibody, respectively, then with Alexa Fluor® 647-labeled goat anti-mouse IgG and its semi-quantitative analysis of mean fluorescence intensity (MFI). Data were shown as mean ± SD. *p <0.05, **p <0.01, ***p <0.001 and ns: not significant.

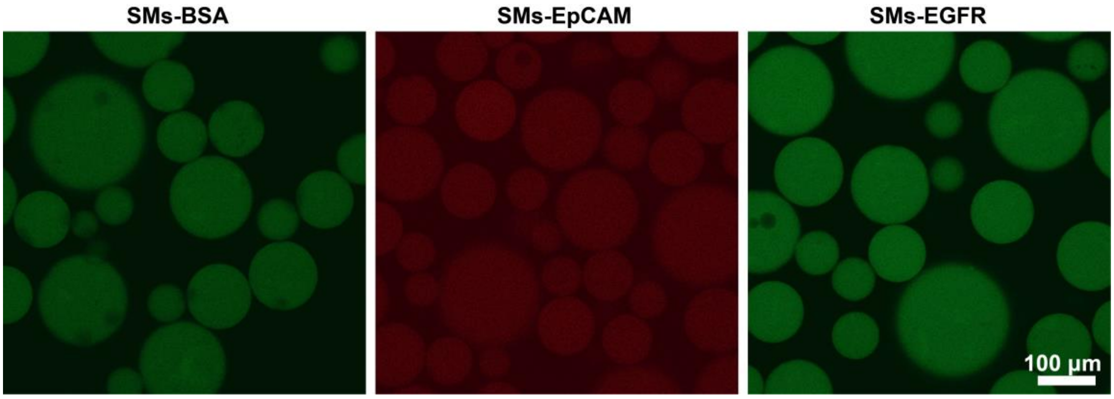

Figure S10. The different functionalized SMs. (FITC-BSA, Alexa Fluor® 647-labeled anti-EpCAM antibody, and FITC-labeled anti-EGFR antibody were used.) Scale bar: 100 μm.

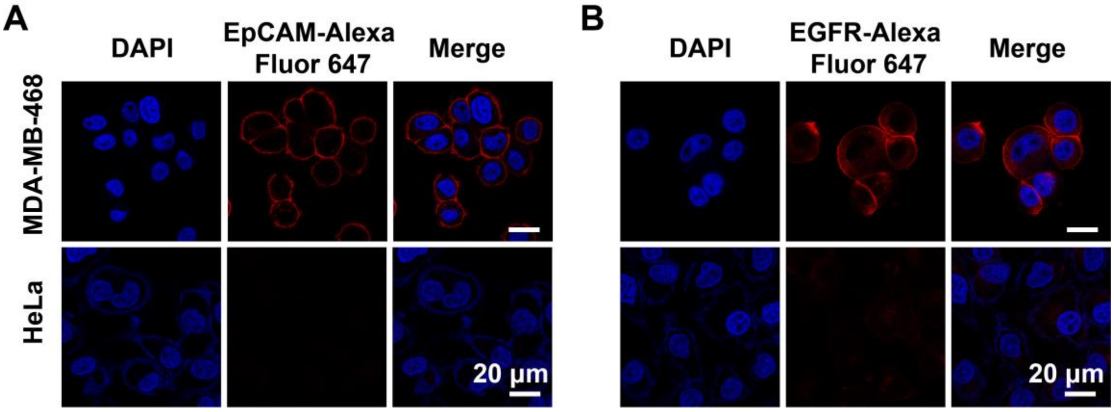

Figure S11. A, B) The expression of EpCAM (the right) and EGFR (the left) in MDA-MB-468 and HeLa cells by immunofluorescence staining. Scale bar: 20 μm.

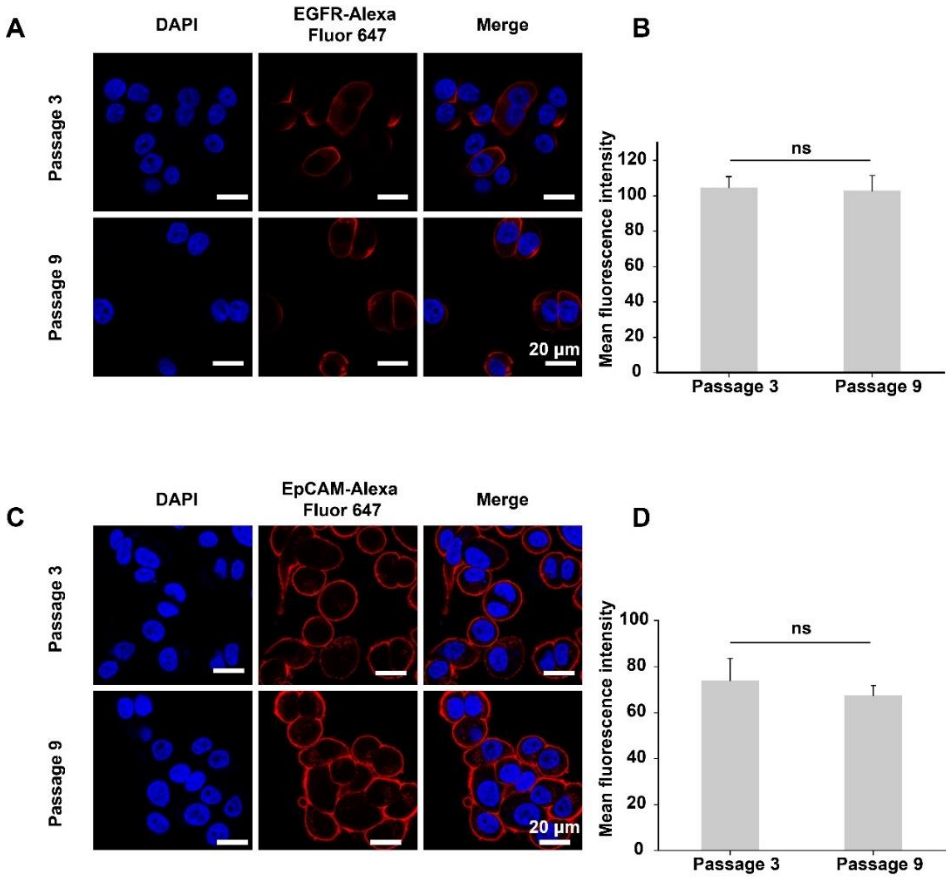

Figure S12. A, B) The EGFR expression of MDA-MB-468 cells (passage3 and passage9) was confirmed by confocal laser scanning imaging and its semi-quantitative analysis by using Image J software (n=3). Scale bar:20 μm. C, D) The EpCAM expression of MDA-MB-468 cells (passage3 and passage9) was confirmed by confocal laser scanning imaging and its semi-quantitative analysis by using Image J software (n=3). Scale bar:20 μm. Data are the mean ± SD. **p* <0.05, ***p* <0.01, ****p* <0.001 and ns: not significant.

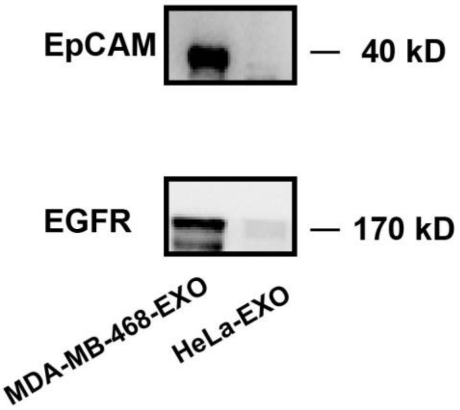

Figure S13. Differential expression of exosomal EpCAM and EGFR in MDA-MB-468 and HeLa cells, respectively.

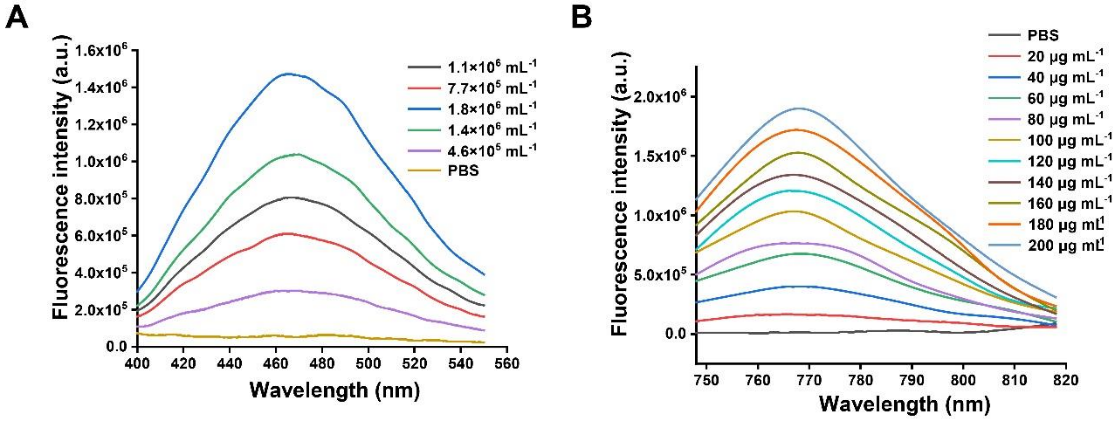

Figure S14. A) Fluorescence spectra of different cell densities of MDA-MB-468 cells prestained with DAPI. B) Fluorescence spectra of different concentrations of MDA-MB-468 derived exosomes prestained with DiR.

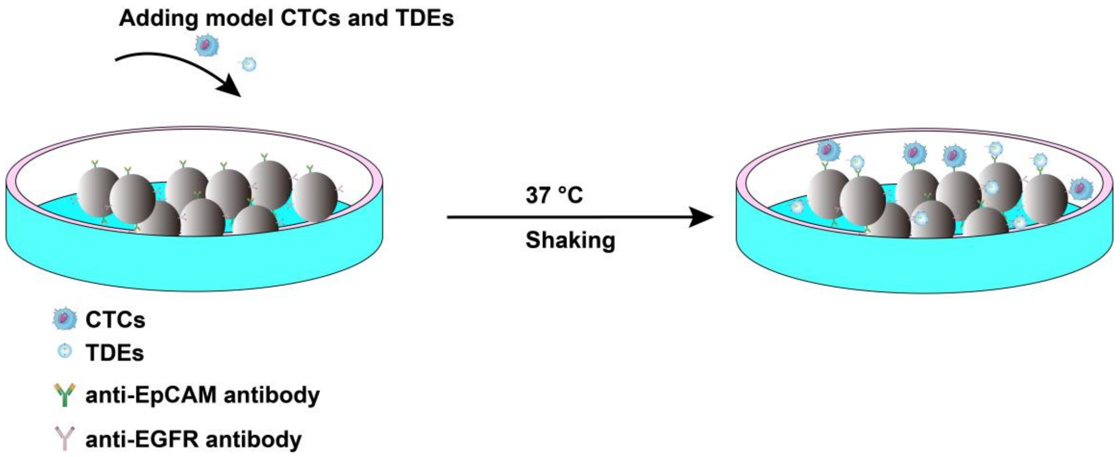

Figure S15. Schematic illustration of the CTCs and TDEs captured under static conditions.

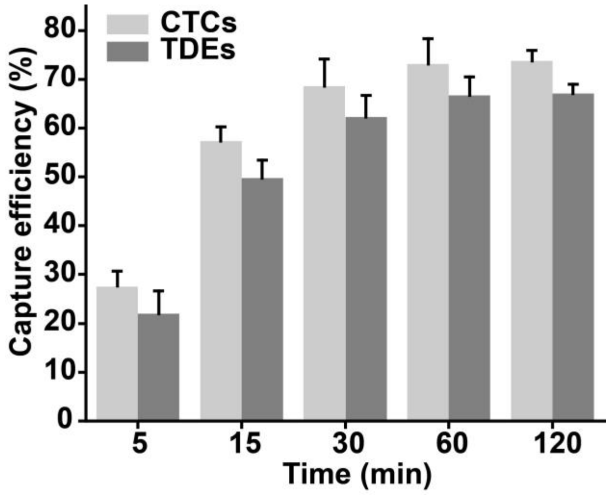

Figure S16. Screening for optimal static capture time (n=3). Data are the mean ± SD.

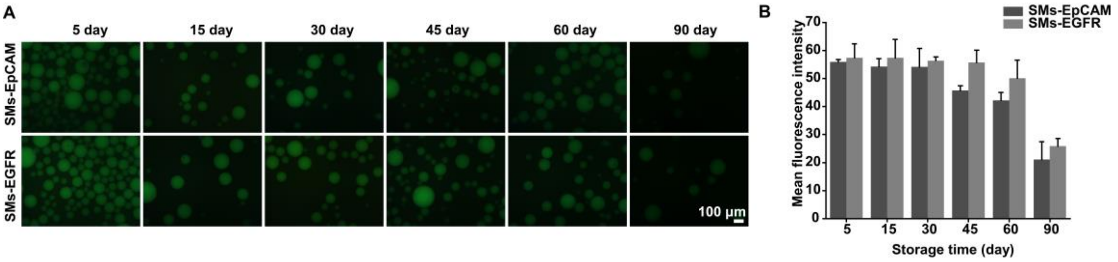

Figure S17. A) Stability of SMs-EpCAM and SMs-EGFR stored at 4°C for 90 days. B) The semi-quantitative analysis by fluorescence intensity (n=3). Data are the mean ± SD.

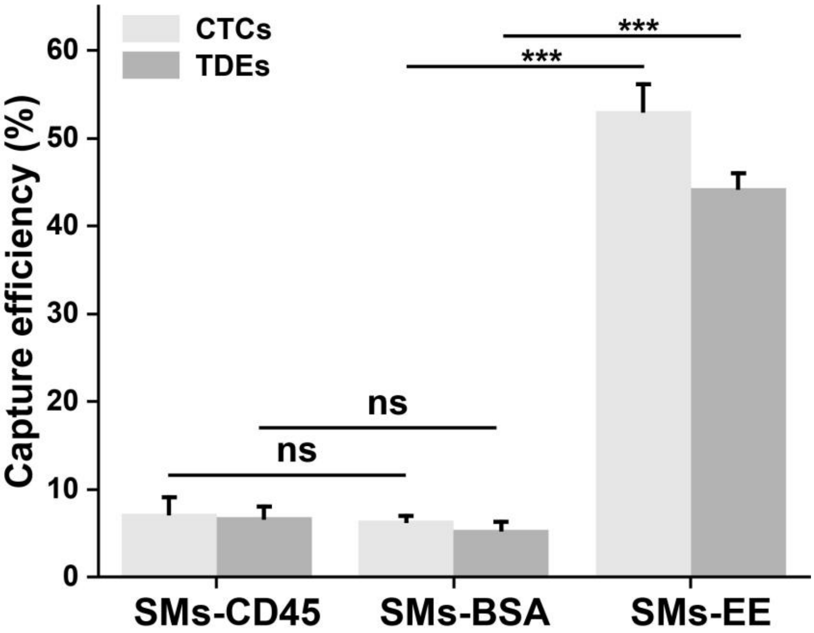

Figure S18. The comparison among SMs-CD45, SMs-BSA, and SMs-EE to capture CTCs and TDEs in a closed-loop circulation system. Data were shown as mean ± SD. *p <0.05, **p <0.01, ***p <0.001 and ns: not significant.

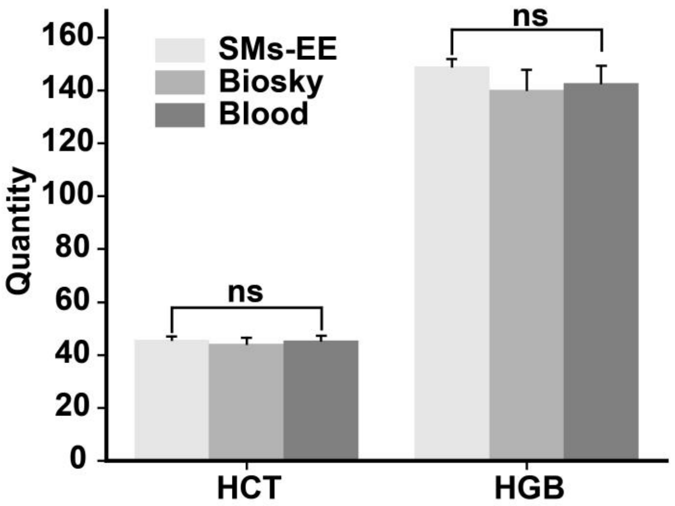

Figure S19. Comparison of the HCT (%) and HGB (g L^{-1}) after incubation of Biosky and SMs-EE in whole blood ($n = 3$, HCT: hematocrit, HGB: hemoglobin). Data are the mean ± SD. *p <0.05, **p <0.01, ***p <0.001 and ns: not significant.

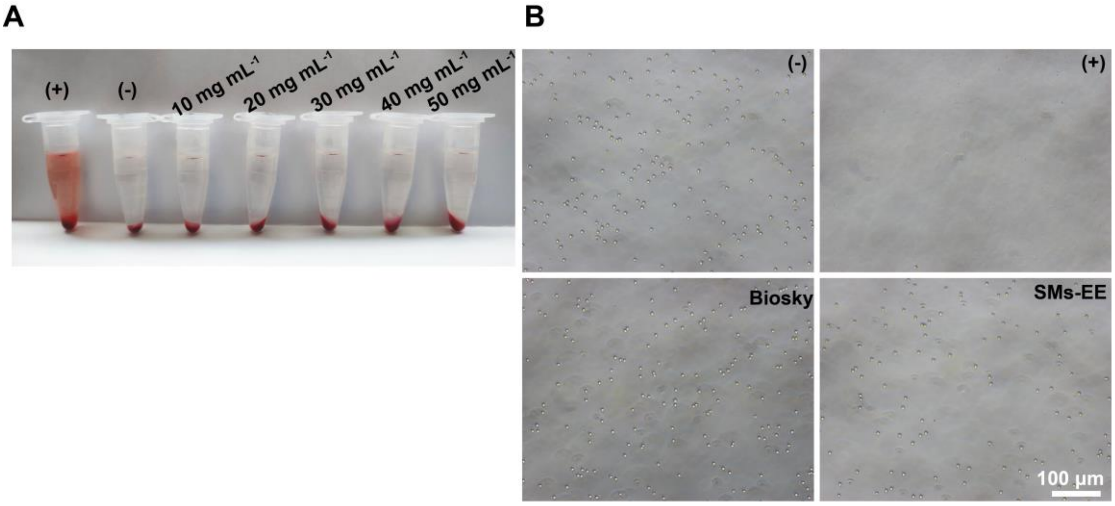

Figure S20. A) Digital photos of the RBC suspensions after incubation with different concentrations of SMs-EE. All the supernatants with different concentrations of SMs-EE were colorless, which indicated that there was no erythrocyte rupture and hemoglobin release. The experiments were performed independently in duplicate with similar results. B) Typical microscopy images showing the morphologies of RBCs. (−): negative control, normal saline. (+): positive control, deionized water. Scale bar: 100 μm.

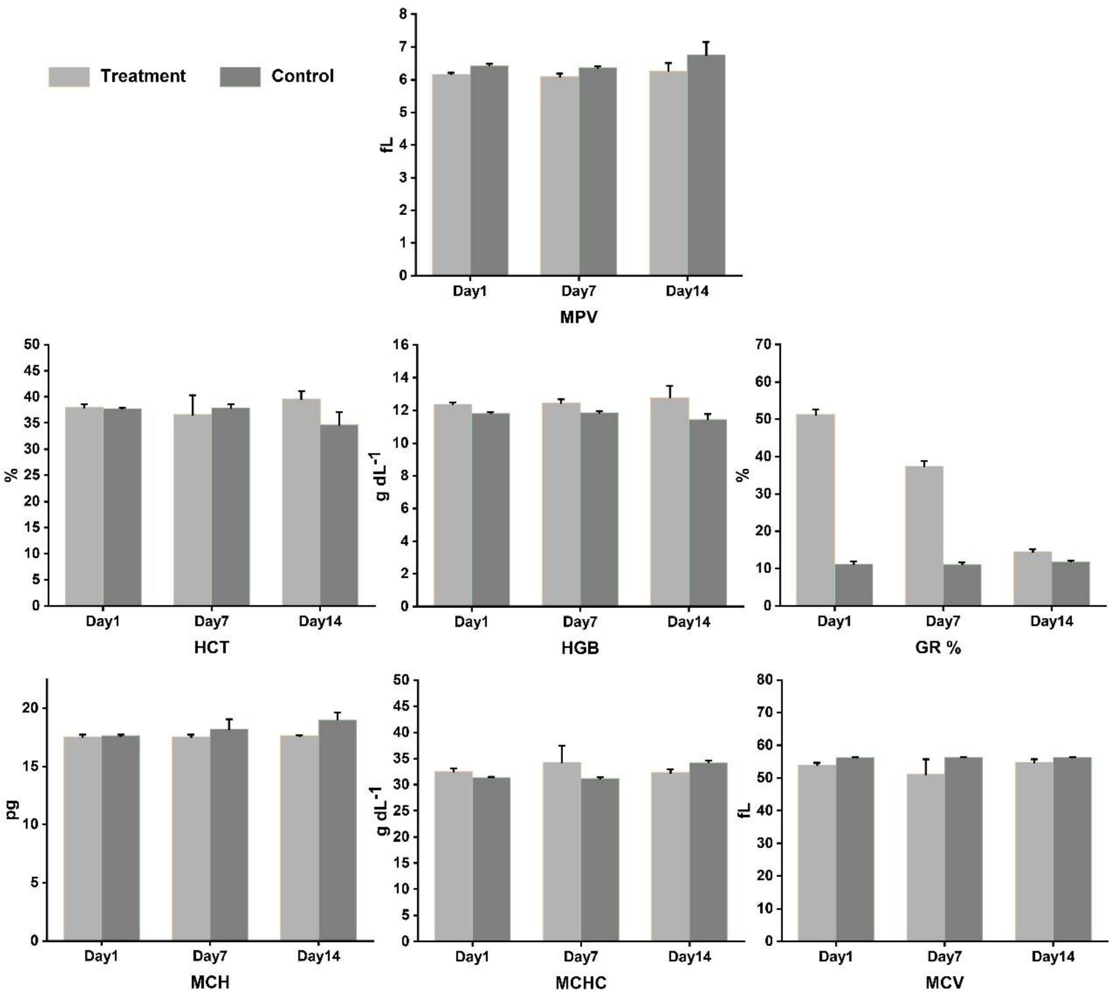

Figure S21. Hematological analysis of the rat after surgery at Day 1, Day 7, and Day14 (n = 3, MPV: mean platelet volume, HCT: hematocrit, HGB: hemoglobin, GR: granulocyte, MCH: mean corpuscular hemoglobin, MCHC: mean corpuscular hemoglobin concentration, MCV: mean corpuscular volume). Data were shown as mean ± SD.

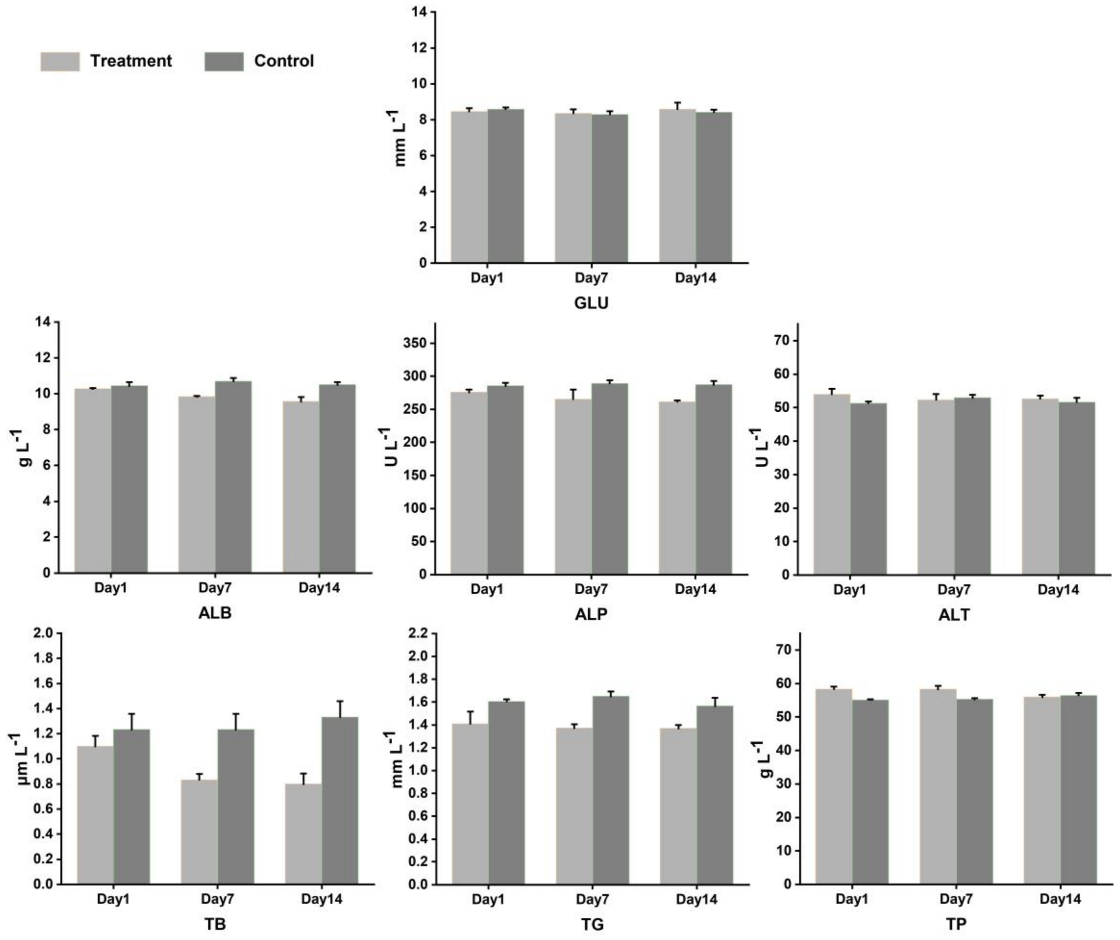

Figure S22. Blood biochemical analysis of the rat after surgery at Day 1, Day 7, and Day14 (n = 3, GLU: glucose, ALB: albumin, ALP: alkaline phosphatase, ALT: alanine amiotransferase, TB: total bilirubin, TG: triglyceride, TP: total protein). Data were shown as mean ± SD.

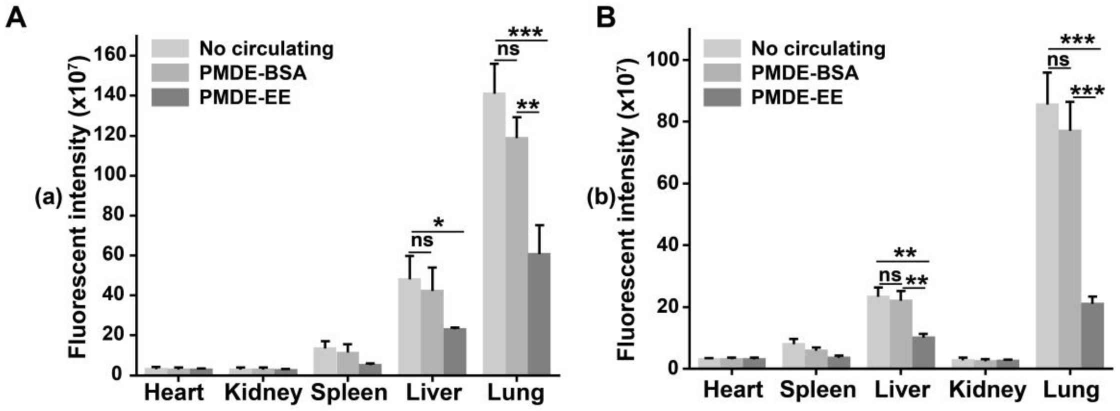

Figure S23. A) ROI analysis of in vitro fluorescence imaging of Balb/c mice at 3h following injection of cancer cells (n=3). B) ROI analysis of in vitro fluorescence imaging of Balb/c mice at 3h following injection of TDEs (n=3). Data were shown as mean ± SD. *p <0.05, **p <0.01, ***p <0.001 and ns: not significant.

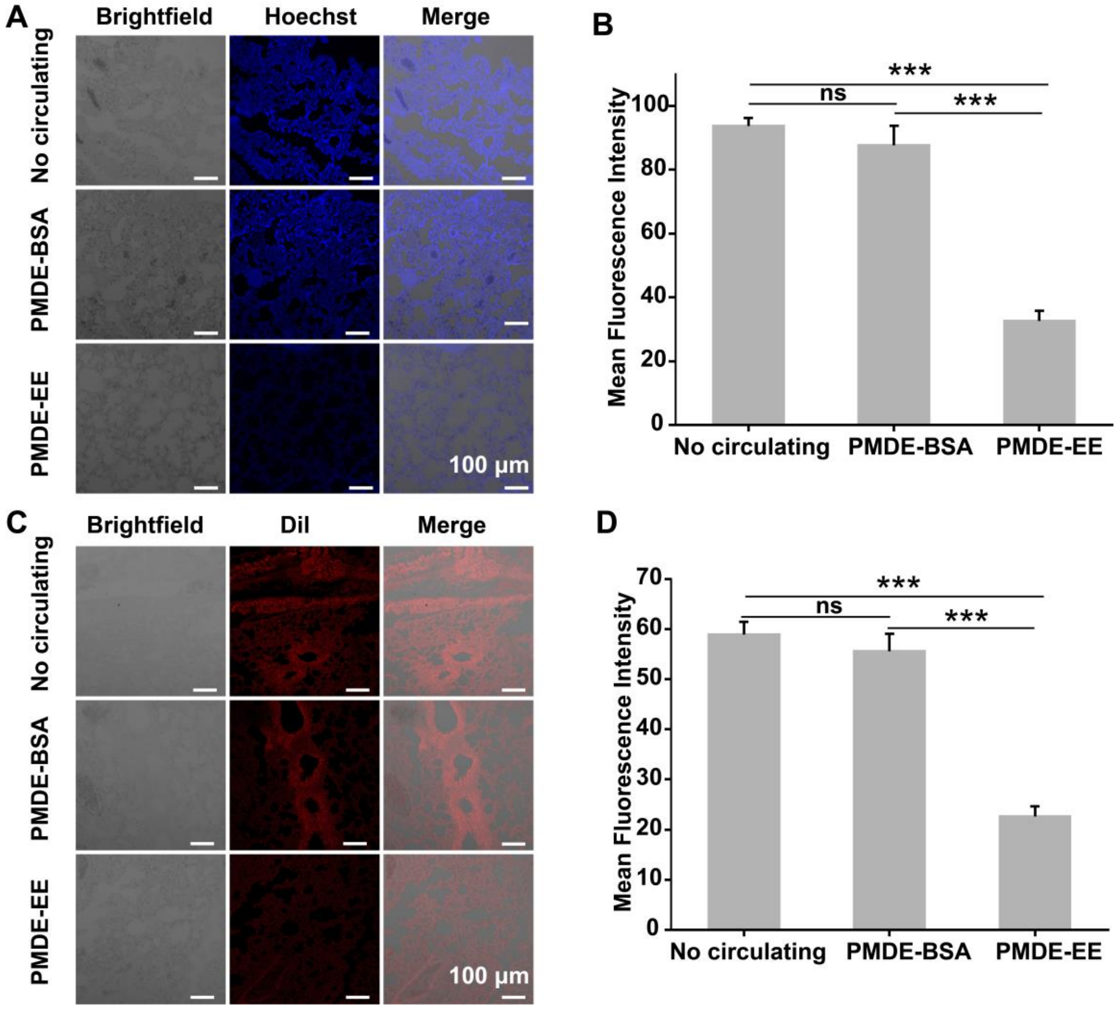

Figure S24. A, B) Representative confocal imaging of the biodistribution of CTCs in the lung of Balb/c mice and its semi-quantitative analysis (n=3). Scale bar: 100 μm. C, D) Representative confocal imaging of the biodistribution of TDEs in the lung of Balb/c mice and its semi-quantitative analysis (n=3). Scale bar: 100 μm. Data were shown as mean ± SD. *p <0.05, **p <0.01, ***p <0.001 and ns: not significant.

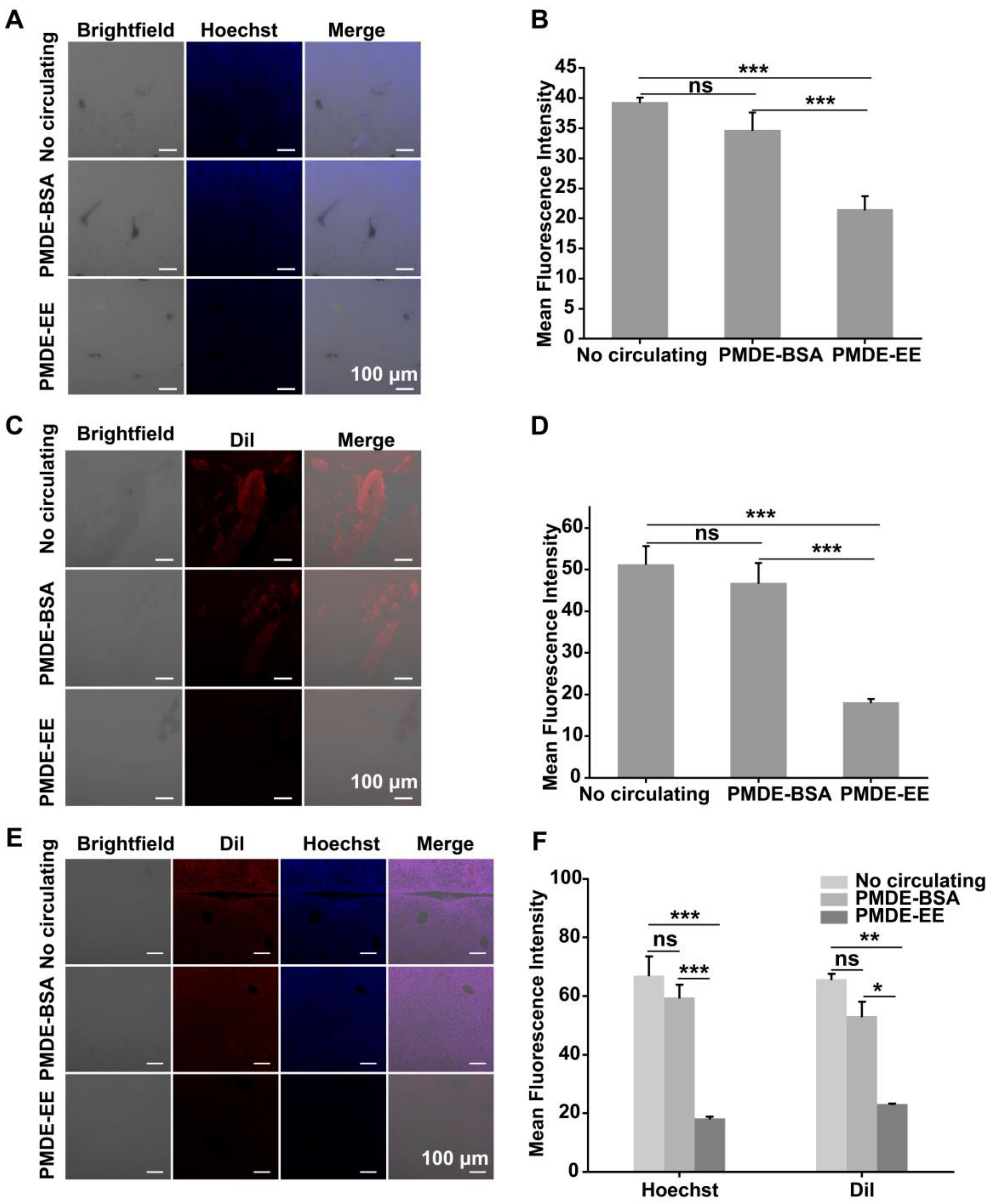

Figure S25. A, B) Representative confocal imaging of the biodistribution of CTCs in the liver of Balb/c mice and its semi-quantitative analysis (n=3). Scale bar: 100 μm. C, D) Representative confocal imaging of the biodistribution of TDEs in the liver of Balb/c mice and its semi-quantitative analysis (n=3). Scale bar: 100 μm. E, F) Representative confocal imaging of the biodistribution of CTCs and TDEs in the liver of Balb/c mice and its semi-quantitative analysis (n=3). Scale bar: 100 μm. Data were shown as mean ± SD. *p <0.05, **p <0.01, ***p <0.001 and ns: not significant.

Table S1. Comparison of our method with other methods for isolating CTCs or TDEs

Separation category subcategory	Method	Capture subject	Selection criteria	Throughput	Sample volume
Immunomagnetic positive enrichment	MagSweeper[3]<br>Mini-SEC[4]	CTCs<br>TDEs	EpCAM<br>CSPG4	9 mL/h	9 mL<br>1 mL
Immunoaffinity	CellSearch[5]<br>Polymer surfaces[6]	CTCs<br>TDEs	EpCAM<br>EpCAM, HER2, EGFR	2.5 mL/h	7.5 mL<br>10 mL
Microfluidic positive immunocapture	HTMSU[7]<br>CTC-Chip[8]<br>GO chip[9]<br>FluidporeFace-Chip[10]	CTCs<br>CTCs<br>CTCs<br>TDEs	EpCAM<br>EpCAM<br>EpCAM<br>EpCAM	1–2 mL/h<br>1–2 mL/h<br>1–3 mL/h<br>30 µL/h	1 mL<br>2.7 mL<br>1 mL<br>100 µL
Negative immunomagnetic enrichment	Negative microfluidic platform[11]	CTCs	CD45	2 mL/h	2 mL
Size-based separation	FMSA[12]<br>Parsortix[13]	CTCs<br>CTCs	8 µm pores<br>4.5-10 µm gap size	45 mL/h<br>10 mL/h	7.5 mL<br>4 mL
N/A	This work	CTCs, TDEs	EpCAM, EGFR	600 mL/h	The entire peripheral blood

Table S2. A comparison of all the currently available methods for in vivo removing CTCs from peripheral blood circulation

Method	Features	Capture subject	Selection criteria	Capture efficiency (in vivo)	Throughput	Whether to validate the effect on biodistribution
MagWIRE[14]	Antibody-coated magnetic particles for labeling CTCs;magnetic wire for intravascular retrieval and enrichment	CTCs	EpCAM	1-8 %	20 mL/min	No
^{HB}GO chip[15]	A microfluidic device for in vivo enrichment of CTCs	CTCs	EpCAM	0.00762%	0.3 mL/min	No
CTC-Net[16]	An injectable and retractable 3-D probe for in vivo intravascular capture of CTCs.	CTCs	EpCAM	3.5%	0.2 mL/min	No
BPNSs-catheter[17]	A black phosphorus and antibody functionalized intravenous catheter;killing CTCs on site.	CTCs	EpCAM	2.1%	5.2 mL/min	No
MPC/NF-catheter[18]	A flexible electronic intravenous catheter; killing CTCs on site.	CTCs	EpCAM	22.3%	5 mL/min	No
HA-MVS[19]	An implantable magnetic vascular scaffold; killing CTCs on site.	CTCs	CD44	5.82-13.26%	5 mL/min	No
This work	Enrichment of CTCs and TDEs from peripheral blood into versatile module in vitro	CTCs, TDEs	EpCAM, EGFR	34.3% and 27.7 %	10 mL/min	Yes

References

[1] L. Sundberg, J. Porath, *J Chromatogr* **1974**, *90*, 87.

[2] C. Thery, S. Amigorena, G. Raposo, A. Clayton, *Curr Protoc Cell Biol* **2006**, *Chapter 3*, Unit 3 22.

[3] G. Deng, S. Krishnakumar, A. A. Powell, H. Zhang, M. N. Mindrinos, M. L. Telli, R. W. Davis, S. S. Jeffrey, *BMC Cancer* **2014**, *14*, 456.

[4] P. Sharma, S. Ludwig, L. Muller, C. S. Hong, J. M. Kirkwood, S. Ferrone, T. L. Whiteside, *J Extracell Vesicles* **2018**, *7*, 1435138.

[5] a) S. Riethdorf, H. Fritsche, V. Muller, T. Rau, C. Schindlbeck, B. Rack, W. Janni, C. Coith, K. Beck, F. Janicke, S. Jackson, T. Gornet, M. Cristofanilli, K. Pantel, *Clin Cancer Res* **2007**, *13*, 920; b) L. Wang, P. Balasubramanian, A. P. Chen, S. Kummar, Y. A. Evrard, R. J. Kinders, *Semin. Oncol.* **2016**, *43*, 464.

[6] M. J. Poellmann, A. Nair, J. Bu, J. K. H. Kim, R. J. Kimple, S. Hong, *Nano Lett* **2020**, *20*, 5686.

[7] a) A. A. Adams, P. I. Okagbare, J. Feng, M. L. Hupert, D. Patterson, J. Gottert, R. L. McCarley, D. Nikitopoulos, M. C. Murphy, S. A. Soper, *J Am Chem Soc* **2008**, *130*, 8633; b) U. Dharmasiri, S. Balamurugan, A. A. Adams, P. I. Okagbare, A. Obubuafo, S. A. Soper, *Electrophoresis* **2009**, *30*, 3289; c) U. Dharmasiri, S. K. Njoroge, M. A. Witek, M. G. Adebiyi, J. W. Kamande, M. L. Hupert, F. Barany, S. A. Soper, *Anal. Chem.* **2011**, *83*, 2301.

[8] S. Nagrath, L. V. Sequist, S. Maheswaran, D. W. Bell, D. Irimia, L. Ulkus, M. R. Smith, E. L. Kwak, S. Digumarthy, A. Muzikansky, P. Ryan, U. J. Balis, R. G. Tompkins, D. A. Haber, M. Toner, *Nature* **2007**, *450*, 1235.

[9] a) H. J. Yoon, T. H. Kim, Z. Zhang, E. Azizi, T. M. Pham, C. Paoletti, J. Lin, N. Ramnath, M. S. Wicha, D. F. Hayes, D. M. Simeone, S. Nagrath, *Nat Nanotechnol* **2013**, *8*, 735; b) H. J. Yoon, A. Shanker, Y. Wang, M. Kozminsky, Q. Jin, N. Palanisamy, M. L. Burness, E. Azizi, D. M. Simeone, M. S. Wicha, J. Kim, S. Nagrath, *Adv Mater* **2016**, *28*, 4891.

[10] Q. Niu, J. Gao, K. Zhao, X. Chen, X. Lin, C. Huang, Y. An, X. Xiao, Q. Wu, L.

Cui, P. Zhang, L. Wu, C. Yang, *Proc Natl Acad Sci U S A* **2022**, *119*, e2213236119.
[11]B. N. Sajay, C. P. Chang, H. Ahmad, P. Khuntontong, C. C. Wong, Z. Wang, P. D. Puiu, R. Soo, A. R. Rahman, *Biomed. Microdevices* **2014**, *16*, 537.
[12]R. A. Harouaka, M. D. Zhou, Y. T. Yeh, W. J. Khan, A. Das, X. Liu, C. C. Christ, D. T. Dicker, T. S. Baney, J. T. Kaifi, C. P. Belani, C. I. Truica, W. S. El-Deiry, J. P. Allerton, S. Y. Zheng, *Clin Chem* **2014**, *60*, 323.
[13]G. E. Hvichia, Z. Parveen, C. Wagner, M. Janning, J. Quidde, A. Stein, V. Muller, S. Loges, R. P. Neves, N. H. Stoecklein, H. Wikman, S. Riethdorf, K. Pantel, T. M. Gorges, *Int J Cancer* **2016**, *138*, 2894.
[14]O. Vermesh, A. Aalipour, T. J. Ge, Y. Saenz, Y. Guo, I. S. Alam, S. M. Park, C. N. Adelson, Y. Mitsutake, J. Vilches-Moure, E. Godoy, M. H. Bachmann, C. C. Ooi, J. K. Lyons, K. Mueller, H. Arami, A. Green, E. I. Solomon, S. X. Wang, S. S. Gambhir, *Nat Biomed Eng* **2018**, *2*, 696.
[15]T. H. Kim, Y. Wang, C. R. Oliver, D. H. Thamm, L. Cooling, C. Paoletti, K. J. Smith, S. Nagrath, D. F. Hayes, *Nat Commun* **2019**, *10*, 1478.
[16]S. B. Cheng, M. Wang, C. Zhang, M. M. Chen, Y. K. Wang, S. Tian, N. Zhan, W. G. Dong, M. Xie, W. H. Huang, *Anal Chem* **2020**, *92*, 5447.
[17]D. Wang, C. Ge, W. Liang, Q. Yang, Q. Liu, W. Ma, L. Shi, H. Wu, Y. Zhang, Z. Wu, C. Wei, L. Huang, Z. Fang, L. Liu, S. Bao, H. Zhang, *Adv Sci* **2020**, *7*, 2000940.
[18]D. Wang, R. Dong, X. Wang, X. Jiang, *ACS Nano* **2022**, *16*, 5274.
[19]Z. Yin, R. Shi, X. Xia, L. Li, Y. Yang, S. Li, J. Xu, Y. Xu, X. Cai, S. Wang, Z. Liu, T. Peng, Y. Peng, H. Wang, M. Ye, Y. Liu, Z. Chen, W. Tan, *Adv Mater* **2022**, e2207870.
